# Human immunomodulatory ligand B7-1 mediates synaptic remodeling via the p75 neurotrophin receptor

**DOI:** 10.1172/JCI157002

**Published:** 2022-11-15

**Authors:** Nicholas C. Morano, Roshelle S. Smith, Victor Danelon, Ryan Schreiner, Uttsav Patel, Natalia G. Herrera, Carla Smith, Steven M. Olson, Michelle K. Laerke, Alev Celikgil, Scott J. Garforth, Sarah C. Garrett-Thomson, Francis S. Lee, Barbara L. Hempstead, Steven C. Almo

**Affiliations:** 1 Department of Biochemistry, Albert Einstein College of Medicine, New York, New York, USA.; 2 Zuckerman Mind Brain Behavior Institute, Columbia University, New York, New York, USA.; 3 Department of Medicine, Weill Cornell Graduate School of Medical Sciences, New York, New York, USA.; 4 Division of Regenerative Medicine, Hartman Institute for Therapeutic Organ Regeneration, Ansary Stem Cell Institute, Department of Medicine, Weill Cornell Medicine, New York, New York, USA.; 5 Department of Computer Science, Georgia Institute of Technology, Atlanta, Georgia, USA.; 6 Department of Psychiatry, Weill Cornell Medicine, New York, New York, USA.

**Keywords:** Neuroscience, Immunotherapy, Neurological disorders, Synapses

## Abstract

Cell surface receptors, ligands, and adhesion molecules underlie development, circuit formation, and synaptic function of the central nervous system and represent important therapeutic targets for many neuropathologies. The functional contributions of interactions between cell surface proteins of neurons and nonneuronal cells have not been fully addressed. Using an unbiased protein-protein interaction screen, we showed that the human immunomodulatory ligand B7-1 (hB7-1) interacts with the p75 neurotrophin receptor (p75^NTR^) and that the B7-1:p75^NTR^ interaction is a recent evolutionary adaptation present in humans and other primates, but absent in mice, rats, and other lower mammals. The surface of hB7-1 that engages p75^NTR^ overlaps with the hB7-1 surface involved in CTLA-4/CD28 recognition, and these molecules directly compete for binding to p75^NTR^. Soluble or membrane-bound hB7-1 altered dendritic morphology of cultured hippocampal neurons, with loss of the postsynaptic protein PSD95 in a p75^NTR^-dependent manner. Abatacept, an FDA-approved therapeutic (CTLA-4–hFc fusion) inhibited these processes. In vivo injection of hB7-1 into the murine subiculum, a hippocampal region affected in Alzheimer’s disease, resulted in p75^NTR^-dependent pruning of dendritic spines. Here, we report the biochemical interaction between B7-1 and p75^NTR^, describe biological effects on neuronal morphology, and identify a therapeutic opportunity for treatment of neuroinflammatory diseases.

## Introduction

Cell surface receptors and ligands coordinate communication, signaling, and functional synergy between cells. These interactions are critical for the integration of complex organ systems and represent important therapeutic targets for pharmacological intervention in a wide range of diseases. B7-1 (CD80) is a canonical costimulatory molecule expressed on antigen-presenting cells (APCs) that directly controls the activation of T cells through interactions with CD28 and CTLA-4 (1). In the settings of aging, central nervous system (CNS) inflammation, injury, and neurodegenerative disease, APCs, such as microglia or infiltrating macrophages, upregulate immunomodulatory proteins, including B7-1. We analyzed the binding interactions of human B7-1 within the Ig and tumor necrosis factor receptor (TNFR) superfamilies and surprisingly identified an interaction with the TNFR superfamily (TNFRSF) member p75 neurotrophin receptor (p75^NTR^, NGFR, TNFRSF16), a major regulator of the RhoA and NF-κB pathways (2).

B7-1 and p75^NTR^ are coexpressed in multiple human organ systems, including the immune system and the nervous system. While B7-1:p75^NTR^ interactions may have broad biological consequences, numerous neurological diseases are characterized by brain infiltration of APCs or by microglia activation and dysregulation. Furthermore, B7-1 expression is documented on activated microglia and APCs, such as brain-infiltrating macrophages, in response to a number of cues, including exposure to LPS and proinflammatory cytokines (3, 4), infections of the brain (5), and neurodegenerative diseases such as multiple sclerosis (MS) (6–8) (Table 1). While evaluation of B7-1 in other conditions has not been systematically examined, induction of peripheral expression of B7-1 is observed in aging (9) and in patients with mild cognitive impairment (10). Therefore, we chose to examine the biological implications of B7-1:p75^NTR^ interactions within the context of p75^NTR^ signaling in the CNS.

p75^NTR^ is constitutively expressed in the CNS throughout development and becomes regionally restricted in adulthood, but is upregulated following injury (11). p75^NTR^ consists of 4 cysteine-rich extracellular domains (CRD1–4) and an intracellular death domain (DD) (12). Activation of p75^NTR^ by the neurotrophin family of ligands (NGF, BDNF, NT3, NT4, proNGF, proBDNF, and proNT3) regulates a myriad of cellular processes (11, 13), including the growth of neuronal processes and the formation and pruning of synapses (14–16) as well as synaptic transmission (17) and cell death (16). These distinct and even opposing actions reflect responses to different ligands and activation of numerous downstream signaling pathways. These include the activation of death effector pathways, utilizing JNK (14), TRAF6 (18), and caspase-3 (19), as well as pathways that acutely alter neuronal structure through RhoA and NF-κB (2, 20). In response to inflammatory conditions or acute injury to the nervous system, such as viral infection, autoimmune disease, and stroke, and in the setting of complex neurological disorders, including Alzheimer’s disease (AD) and experimental autoimmune encephalitis (EAE), p75^NTR^ is upregulated and may adversely affect neuronal function (15, 21).

Here, we identify the specific interaction between human immunomodulatory ligand B7-1 (hB7-1) and p75^NTR^, members of the immunoglobin and TNFR superfamilies, respectively. We demonstrate that the hB7-1:p75^NTR^ interaction occurs in primates, but not in rodents, which likely reflects the low (35%–40%) sequence identity of B7-1 in rodents and primates. However, human B7-1 binds rodent p75^NTR^, as p75^NTR^ is highly conserved across species. We define the biochemical determinants responsible for the hB7-1:p75^NTR^ interaction and compare these to the determinants that confer binding to other partners (CD28, CTLA-4) of hB7-1 using mutagenesis. Using cultured murine hippocampal neurons, we demonstrate that hB7-1 is a potent agonist for p75^NTR^ and observe acute p75^NTR^-mediated deleterious remodeling of neuronal dendritic morphology, similar to that elicited by proneurotrophins. We further show that administration of abatacept (Orencia), an FDA-approved drug for rheumatoid arthritis consisting of the extracellular domain of CTLA-4 fused to human Fc, abrogates these effects by inhibiting B7-1:p75^NTR^ binding. Finally, by injecting hB7-1–Fc into the subiculum of mice, we demonstrate that synaptic elimination occurs in vivo in a hB7-1:p75^NTR^–dependent manner. Collectively, these findings broaden our understanding of how proinflammatory states negatively affect neuronal structure and demonstrate mechanisms by which APCs can affect neuronal structure. These observations expand the range of molecular mechanisms by which p75^NTR^ may regulate neuronal function and suggest potential strategies for treating neuroinflammatory degenerative diseases.

## Results

### hB7-1 directly interacts with p75^NTR^.

**Figure 1:**
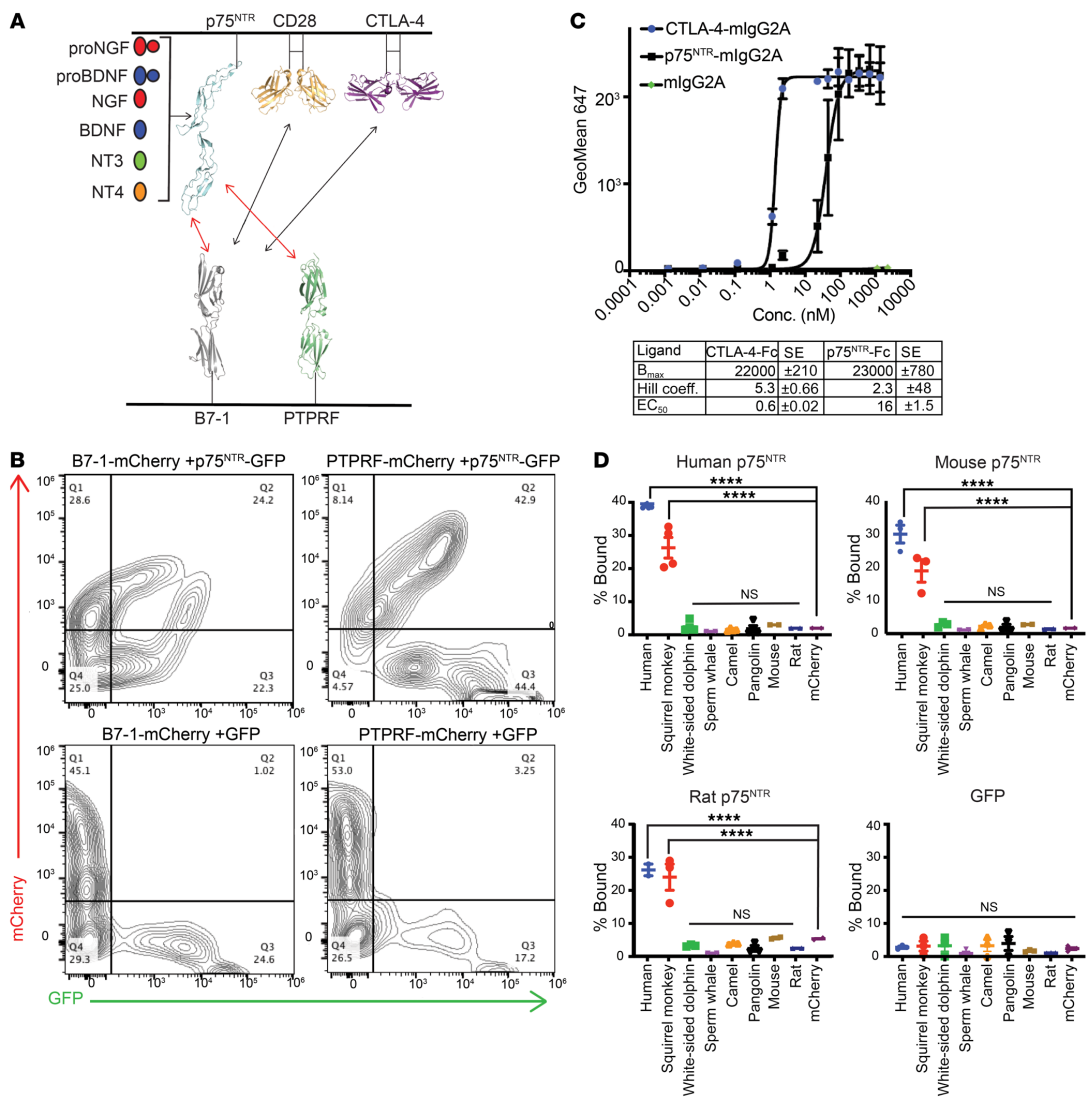
Biochemical and phylogenetic characterization of B7-1:p75^NTR^ interactions. (**A**) Schematic indicating the results of high-throughput cell-cell screening of B7-1 and p75^NTR^ against 395 members of the human Ig and TNFR superfamilies. B7-1–expressing cells bound to cells expressing CD28, CTLA-4, and p75^NTR^, but not cells expressing PD-L1. p75^NTR^–expressing cells bound to cells expressing B7-1 and PTPRF. (**B**) Representative flow plots demonstrating that hB7-1–mCherry cells and PTPRF-mCherry cells bind to cells expressing p75^NTR^-GFP cells, but not GFP control cells. (**C**) Binding between B7-1 and p75^NTR^ was validated by recombinant protein titrations of p75^NTR^ -mIgG2A and CTLA-4–mIgG2A onto streptavidin beads coated with B7-1–hIgG1 biotin. Binding was detected using an anti-mIgG2A antibody, flow cytometry and binding curves were generated, and B_max_, EC_50_, and Hill coefficient (coeff.) were calculated using the equation *Y* = *B_max_* × *X^h^*/(*Kd^h^* + *X^h^*). *n* = 2–3. Error bars represent SEM. (**D**) Cells expressing B7-1–mCherry from various mammalian species were screened against human p75^NTR^-GFP–expressing cells (top left, *n* = 2–4), mouse p75^NTR^-GFP–expressing cells (top right, *n* = 2–3), rat p75^NTR^-GFP–expressing cells (bottom left *n* = 2–3), or cells expressing only GFP (bottom right, *n* = 3), indicating that B7-1:p75^NTR^ interactions are conserved in primates, but not mice. ****P < 0.0001, 1-way ANOVA with multiple comparisons. Each assay represents the indicated number of independent experiments, which each include a single replicate. For mouse B7-1, rat B7-1, and mCherry cells alone, only 2 independent experiments were conducted, each with 2 biological replicates.

To identify binding partners of hB7-1, we utilized a screening strategy in which cells expressing human B7-1–mCherry fusion protein were used to probe cells individually expressing a library of 395 members of the human Ig and TNFR superfamilies tagged with a C-terminal GFP (22). This approach revealed transinteractions of hB7-1 with CTLA-4 and CD28, as expected, and a previously uncharacterized interaction with p75^NTR^ (TNFRSF16) (Figure 1, A and B, Supplemental Figure 1, A–C, and Supplemental Table 1; supplemental material available online with this article; https://doi.org/10.1172/JCI157002DS1). Binding of hB7-1–expressing cells to PD-L1–expressing cells was not observed, as the B7-1:programmed death-ligand 1 (PD-L1) interaction occurs in *cis* (1, 23, 24). p75^NTR^-expressing cells were then screened against the same library, and again, B7-1 binding as well as an additional interaction with protein tyrosine phosphatase receptor type F (PTPRF) was observed (Figure 1, A and B, Supplemental Figure 1, B and C, and Supplemental Table 1). No binding was observed between hB7-1 and other members of the TNFR superfamily (e.g., TNFR2, HVEM, FAS, etc.) or between p75^NTR^ and other members of the human B7 family (e.g., B7-2, ICOSL, PD-L1, etc.) (Supplemental Table 1). To confirm that hB7-1:p75^NTR^–mediated cell conjugation is the result of direct contacts between B7-1 and p75^NTR^, recombinant biotinylated B7-1–Fc protein (for conjugation to streptavidin beads), CTLA-4–Fc, and p75^NTR^-Fc were purified (Supplemental Figure 4) and B7-1:p75^NTR^ binding was assessed using an in vitro flow cytometry–based titration assay. Flow cytometry titrations demonstrated an approximately 30-fold higher EC_50_ of B7-1:p75^NTR^ (16 nM) compared with B7-1:CTLA-4 (0.6 nM) using recombinant proteins (Figure 1C). Hill coefficients of 2.3 for B7-1:p75^NTR^ binding and 5.3 for B7-1:CTLA-4 were observed, suggesting cooperativity; however, these results were obtained using dimeric fusion proteins and may not reflect the behavior of native proteins in the plasma membrane.

While p75^NTR^ sequences are highly conserved in mammals and many vertebrates (25), B7-1 sequences exhibit greater variability (Supplemental Figure 2, A and B). Therefore, mammalian B7-1 sequences with varying degrees of sequence conservation were screened against p75^NTR^ orthologues from human, mouse, and rat. While human and old-world monkey B7-1 bound human p75^NTR^, no interaction was observed with the other B7-1 orthologues examined, including those from sperm whale (the closest nonprimate B7-1 sequence examined) and the commonly used laboratory model organisms, mice and rats (Figure 1D). However, human/primate B7-1 bound mouse or rat p75^NTR^, likely due to high sequence conservation of p75^NTR^ across mammalian species (Figure 1D). Notably, rodent B7-1 (rat or mouse) does not interact with rodent p75^NTR^, suggesting that the interaction may be a recent evolutionary adaptation.

### The p75^NTR^-binding site on B7-1 overlaps with the binding sites of CD28 and CTLA-4, but not PD-L1.

**Figure 2:**
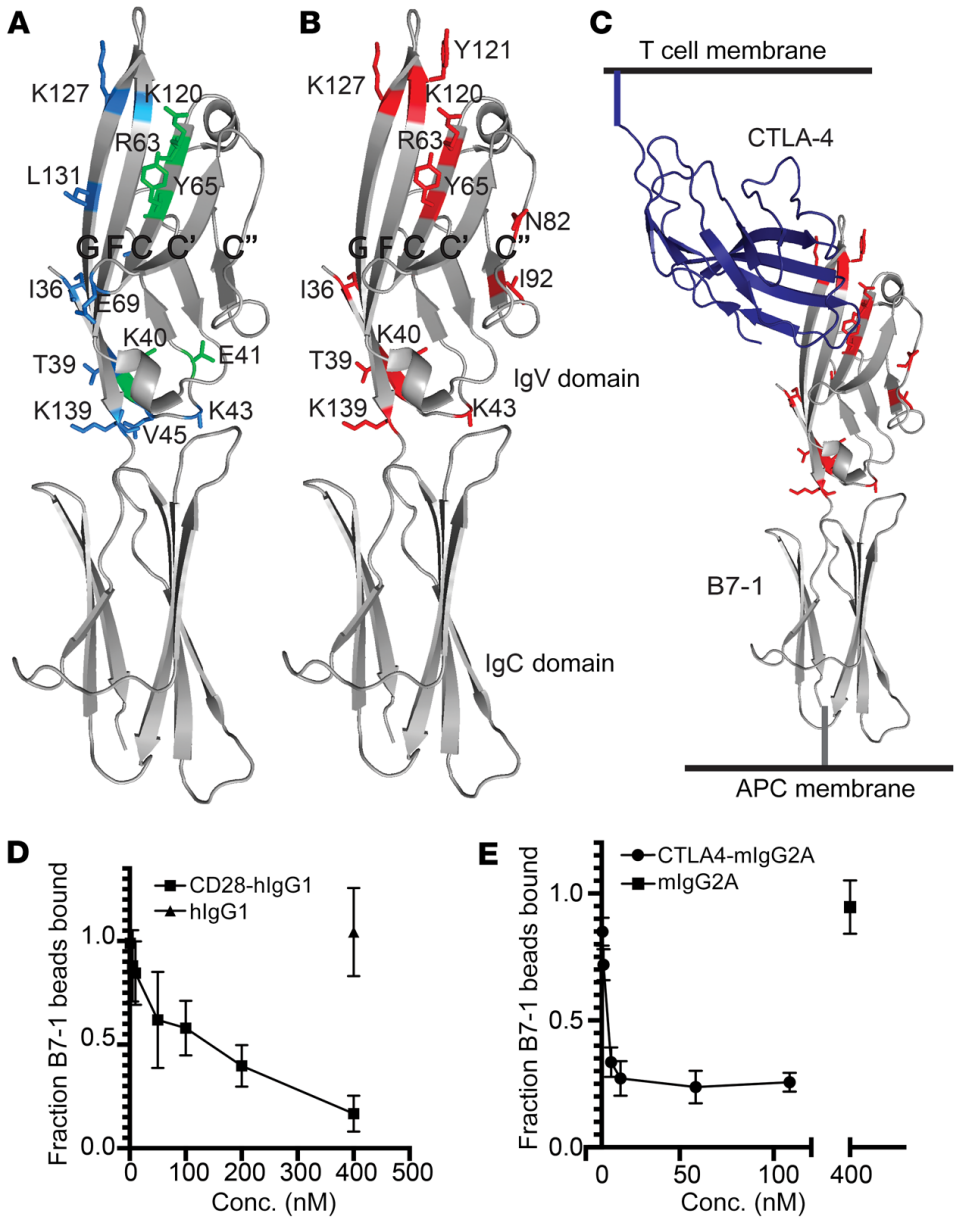
p75^NTR^:hB7-1 interactions depend on the GFCC′C′′ face of hB7-1 and directly compete with CD28 and CTLA-4. (**A**) hB7-1 residues that when mutated showed more than 25% losses in binding to CD28 (blue) or both CD28 and CTLA-4 (green) mapped onto crystal structure of hB7-1 (PDB: 1I8L) (**B**) hB7-1 residues that when mutated showed more than 25% losses in binding to p75^NTR^ (red) mapped onto crystal structure of hB7-1 (PDB: 1I8L). (**C**) Same residues as in **B**, except with monomer of CTLA-4 shown. (**D**) CD28-hIgG1 competes for binding to B7-1 with p75^NTR^. *n* = 6. (**E**) CTLA-4–mIgG2A competes for binding to B7-1 with p75^NTR^. *n* = 3. Each assay represents the indicated number of independent experiments, which each include a single replicate. Error bars represent SEM.

To map the surfaces involved in forming the B7-1: p75 complex, we utilized a strategy in which solvent accessible residues are systematically changed to alanine, aspartic acid, or arginine; we previously employed this approach to generate structural information about transmembrane receptor/ligand complexes (24, 26–28). The surface on B7-1 that engages p75^NTR^ was defined using a library of HEK293F cells individually expressing 92 B7-1 variants harboring mutations in the IgV domain, where solvent accessible residues were mutated to alanine, aspartic acid/glutamic acid, or arginine (Supplemental Figure 3, A and C, and Supplemental Table 2). B7-1 mutants expressed on HEK293F cells were assayed for their ability to bind HEK293F cells expressing CTLA-4–GFP, CD28-GFP, or p75^NTR^-GFP. In total, 18 mutations exhibited decreased binding to 1 or more binding partner, and 11 showed decreased binding to p75^NTR^ (Figure 2, A and B, and Supplemental Table 2). The B7-1 mutants I36A, I36D, T39A, K40D, E41A, K43D, V45D, S49A, R63D, Y65A, E69A, K120D, K127D, L131D, and K139D resulted in a greater than 25% loss in binding to CD28 or both CD28 and CTLA-4 (Figure 2A and Supplemental Table 2). The majority of these mutations (all except for T39A, V45D, S49A, and K139D) are either directly consistent with the CTLA-4–binding site observed in the crystal structure of the CTLA-4:B7-1 complex or are present on the GFCC′C′′ face of B7-1 (Fig 2A and Supplemental Table 2). Twelve mutations, I36D, T39A, Y40D, K43D, S49A, R63D, Y65A, N82E, K120D, Y121D, K127D, and K139D, decreased binding to p75^NTR^ by more than 25% (Figure 2B). Of these, N82E, I92D, and Y121D specifically caused losses in binding to p75^NTR^, but not to CTLA-4 or CD28. Mapping these 12 B7-1 mutations onto the crystal structure of the B7-1:CTLA-4 complex demonstrated clustering at and near the CTLA-4–binding site on the GFCC′C′′ face of B7-1 (Figure 2, B and C). However, p75^NTR^ also recognizes residues on the C′′ strand of B7-1 that are not involved in CTLA-4/CD28 binding. N82, located in the middle of the C′′ strand, and Y121, located at the top of the F strand of B7-1, are both outside the putative CTLA-4/CD28-binding site. I92 is located on the dimerization surface of B7-1 and directly behind N82 on the C′′ strand, and mutation of this residue may cause perturbations to the C′′ strand that alter p75^NTR^ binding. These results suggest that the B7-1 recognition surfaces for CTLA-4, CD28, and p75^NTR^ physically overlap and that the p75^NTR^ recognition surface is more expansive than that of CD28 and CTLA-4 (Figure 2C).

We additionally evaluated the PD-L1–binding surfaces on B7-1. Recent reports indicate that cells expressing B7-1 do not bind cells expressing PD-L1 and that B7-1 binds to PD-L1 in *cis* on the same cell surface (1, 23, 24). Because of this constraint, the PD-L1–binding site was mapped onto human B7-1 using recombinant human PD-L1–Fc, which can be appropriately presented for productive binding. We incubated 1 μM PD-L1–Fc with cells expressing our library of B7-1 point mutations and detected B7-1:PD-L1 binding by flow cytometry. Eleven B7-1 mutants (S49A, N53A, N53D, S55A, E57R, N89A, N89D, I92A, I92D, A104D, K139A) had greater than 70% losses in binding to PD-L1–Fc compared with WT B7-1 (Supplemental Figure 5). These residues are distinct from the p75^NTR^ recognition site and consistent with previous data from our group and others demonstrating that the PD-L1– and CTLA-4/CD28-binding sites are located on opposite faces of the human B7-1 IgV domain (1, 24). Of the 18 mutants with decreased binding to either CTLA-4, CD28, or p75^NTR^, 16 bound PD-L1 similarly to WT B7-1 (Supplemental Figure 5B and Supplemental Table 2). Overall, these results suggest that on B7-1, the PD-L1–binding site and p75^NTR^–binding site are distinct.

To confirm that the B7-1:p75^NTR^–binding interface overlaps with the CD28- and CTLA-4–binding interfaces, in vitro ligand competition experiments were performed. B7-1–Fc was conjugated to fluorescent protein A beads, which bound to HEK293 cells expressing p75^NTR^ (detected using flow cytometry). Titration of increasing concentrations of CTLA-4–Fc or CD28-Fc interfered with B7-1:p75^NTR^ binding, while isotype control protein did not (Figure 2, D and E). These results are consistent with the shared/overlapping binding interfaces for CD28, CTLA-4, and p75^NTR^ suggested by the mutagenesis mapping data. CTLA-4–Fc was more effective than CD28-Fc at inhibiting B7-1:p75 binding, likely due to the reported approximately 10-fold higher affinity of CTLA-4 for B7-1 than CD28 (29).

To further validate our model of B7-1 binding to p75^NTR^, a recombinant soluble form of a B7-1 point mutant (N82E) that specifically lost binding to p75^NTR^, but that maintained near WT binding to CTLA-4, CD28, and PD-L1, was purified (N82E; Figure 2 and Supplemental Figures 4 and 6). We titrated B7-1–Fc and B7-1N82E–Fc against cells expressing CTLA-4, CD28, or p75^NTR^ and calculated EC_50_s for each. B7-1–Fc bound to cells expressing either CD28, CTLA-4, or p75^NTR^. While B7-1N82E–Fc bound CTLA-4– and CD28-expressing cells similarly to B7-1–Fc, binding between B7-1N82E-Fc and p75^NTR^-expressing cells could not be detected (Supplemental Figure 6, A–F), confirming an important role for this residue in p75^NTR^ binding.

### The binding site of B7-1 on p75^NTR^ partially overlaps with the neurotrophin-binding site.

**Figure 3:**
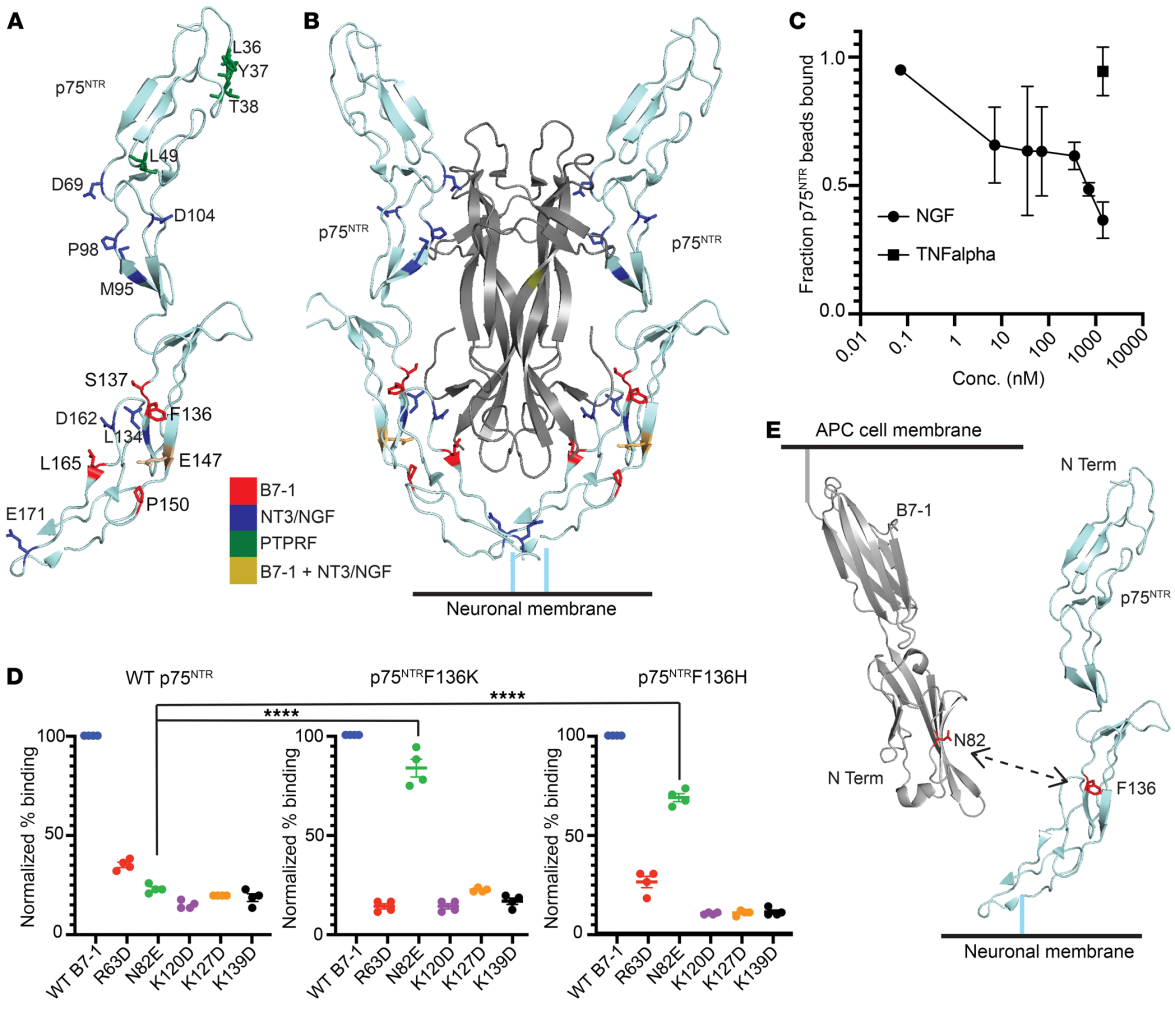
Identification of the binding interface of hB7-1 and PTPRF on p75^NTR^ using epitope- mapping and ligand-competition experiments. (**A**) p75^NTR^ residues identified as important for hB7-1 binding mapped onto crystal structure of rat p75^NTR^ bound to NT3 (PDB: 3BUK) affect binding to B7-1 (red), make contacts with NT3 and also affect binding to B7-1 (brown), and make H bond contacts with NT3 (blue). (**B**) The hB7-1–binding site modeled onto the dimeric structure of p75^NTR^ bound to an NT3 dimer (PDB: 3BUK). (**C**) Ligand-competition experiments showing that NGF and hB7-1 compete for binding to p75^NTR^ at high concentrations. *n* = 3. (**D**) Cells expressing p75^NTR^ -F136K and F136H demonstrate binding to B7-1N82E–expressing cells as well as WT B7-1–expressing cells. ****P < 0.0001, 1-way ANOVA with multiple comparisons. *n* = 4. (**E**) Crystal structures of hB7-1 and p75^NTR^ indicating proposed interaction between p75^NTR^-F136 and B7-1–N82. Each assay represents the indicated number of independent experiments, which each include a single replicate. Error bars represent SEM.

The binding site of B7-1 on p75^NTR^ was mapped using the same strategy employed to define binding surfaces on B7-1. Solvent accessible residues of the p75^NTR^ extracellular domain (calculated using GetArea; ref. 30) were mutated to alanine, aspartic acid/glutamic acid, or arginine in the context of a construct encoding full-length p75^NTR^ fused at the cytoplasmic C-terminus to GFP. We identified 80 residues and successfully generated 114 p75^NTR^-GFP point mutants. This library was screened against B7-1–Fc to identify point mutations that affected binding (Figure 2A, Supplemental Figure 2, B and D, and Supplemental Table 3). To ensure the structural integrity of the p75^NTR^ point mutants, we also screened the library against cells expressing PTPRF-mCherry, which also interacts with p75^NTR^ (Figure 1B). In total, 8 point mutations covering 7 residues demonstrated greater than 50% losses in binding to B7-1 (F136D, S137A, S137D, E147D, P150A, P150D, L165A, and R182A) (Figure 3A). Of these mutations, only 1 (R182A) affected binding to PTPRF, confirming the overall structural integrity of the other 7 mutations. Mutations that affected binding to B7-1 mapped to the more membrane-proximal region of p75^NTR^ (CRD3 and CRD4 domains), and one of these mutations (E147A) overlapped with the neurotrophin binding site, as determined by x-ray crystallography (13) (Figure 3, A and B). These data suggest that the surfaces on p75^NTR^ that interact with both B7-1– and neurotrophin-binding sites only modestly overlap. While NGF at micromolar concentration was able to compete with B7-1 binding, this is unlikely to be biologically relevant, as NGF is present in adult human brain at subnanomolar concentrations (31) (Figure 3C).

To further confirm the results of our mutagenesis data and to provide additional molecular detail about the B7-1:p75^NTR^–binding interface, a modest screen was conducted for “salt bridge suppressors” that restored binding to defective mutants. p75^NTR^ residues of apparent importance for B7-1 binding (F136, S137, P150, L165, P166, and R182) were mutated to both lysine and histidine. Cells expressing these point mutations were screened against cells expressing aspartic and glutamic acid B7-1 point mutants that lost binding to WT p75^NTR^ (R63, N82, K120, K127, and K139). Binding to B7-1 N82E was rescued by both p75^NTR^ F136K and F136H, but not any other p75^NTR^ lysine or histidine mutations (Figure 3D and Supplemental Figure 6G). None of the other B7-1 D/E mutants bound to any p75^NTR^ K/H mutants examined (Supplemental Figure 6, G–I). These observations suggest that N82 on B7-1 is in proximity to F136 on p75^NTR^ and are consistent with our proposed binding interface, which partially overlaps with the neurotrophin-binding site (Figure 3E).

### B7-1 is a p75^NTR^ agonist that disassembles dendritic spines through a p75^NTR^-dependent mechanism.

**Figure 4:**
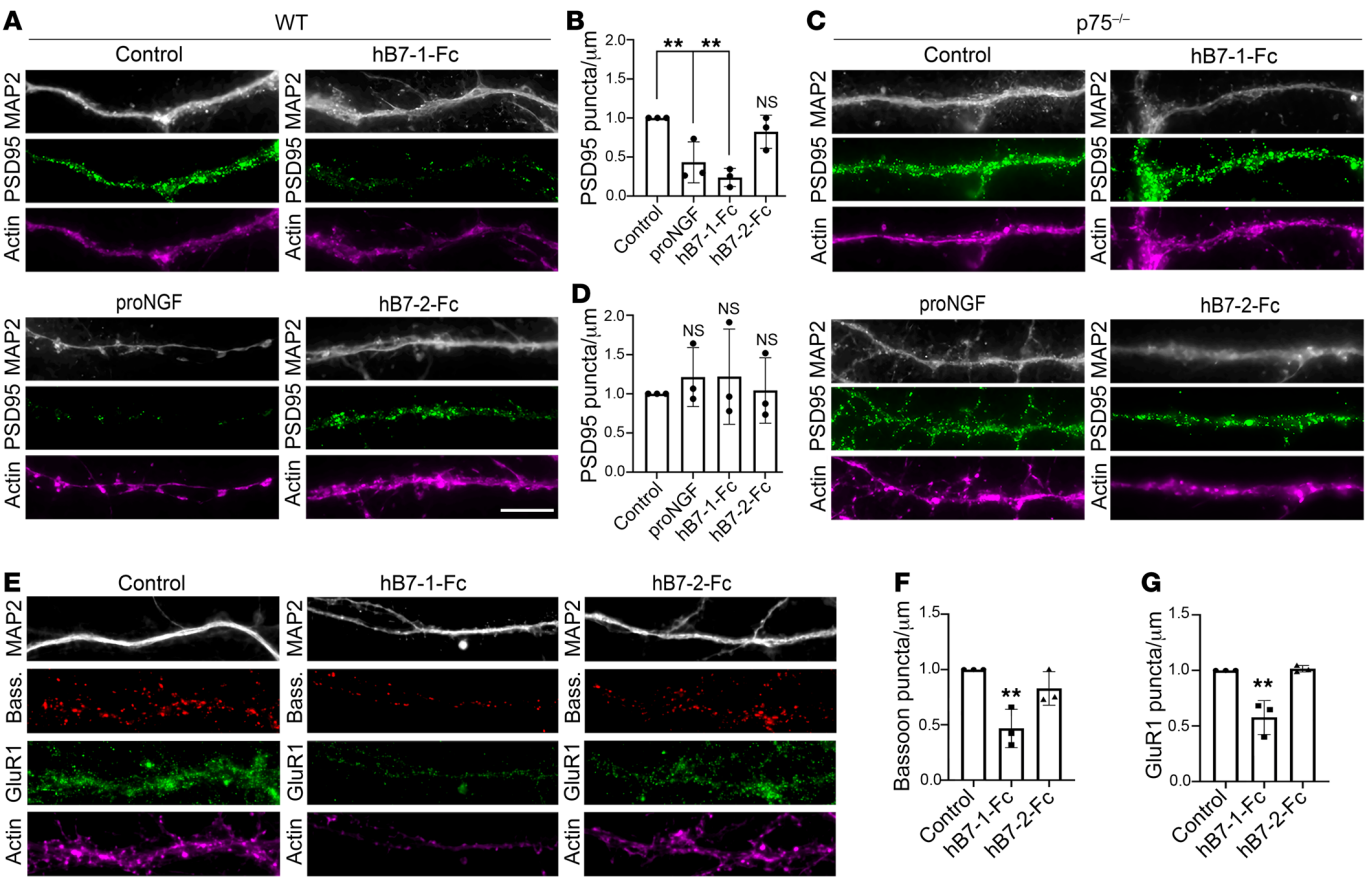
Recombinant hB7-1–Fc induces synapse remodeling similarly to proNGF. (**A**) Representative IF images of WT hippocampal neurons treated for 2 hours with proNGF (10 nM), hB7-1–Fc (750 nM), or hB7-2–Fc (750 nM) and stained for actin, PSD95, and MAP2. (**B**) Quantification of PSD95 puncta density with indicated treatments. Recombinant hB7-1–Fc but not hB7-2–Fc induced a decrease in PSD95 density in cultured neurons, similarly to proNGF treatment. (**C**) Representative IF images of *p75^–/–^* hippocampal neurons treated with proNGF (10 nM), hB7-1–Fc (750 nM), or hB7-2–Fc (750 nM) and stained for actin, PSD95, and MAP2. (**D**) Quantification of PSD95 puncta density with indicated treatment. Recombinant hB7-1–Fc failed to induce a decrease in PSD95 density in cultured neurons. (**E**) Representative IF images of WT hippocampal neurons treated with hB7-1–Fc (750 nM) or hB7-2–Fc (750 nM) and stained for actin, GluR1, bassoon, and MAP2. (**F**) Quantification of bassoon puncta density with indicated treatment. (**G**) Quantification of GluR1 puncta density with indicated treatment. **P < 0.01, 1-way ANOVA with Dunnett’s multiple-comparisons test. *n* = 3 independent experiments. Experiments under each condition were performed in triplicate, and 13 to 15 dendritic segments were analyzed per condition. Scale bar: 20 μm. Error bars represent SEM. See also Supplemental Figure 7.

B7-1 is known to be expressed on APCs, specifically microglia and brain-infiltrating macrophages (Supplemental Table 1), in the setting of CNS inflammation and injury, and p75^NTR^ is similarly induced by CNS neurons in acute injury models. Thus, we examined the effect of B7-1 on mature, cultured p75^NTR^-expressing hippocampal neurons (Figure 4 and Supplemental Figure 7). Our previous work (32) demonstrated that pro forms of neurotrophins and a variant form of the BDNF prodomain can induce acute dendritic spine collapse, as detected by a transition from mushroom spines to filopodia-like protrusions, relocalization of PSD95 from the spine head to the dendritic shaft, and a concomitant significant decline in the number of apposed presynaptic sites, as detected by anti-bassoon antibody. This spine collapse involves signaling through actin cytoskeletal regulators downstream of p75^NTR^, which is localized to postsynaptic sites in the hippocampus in vivo and in cultured hippocampal neurons (Figure 4). To assess the contribution to spine collapse, we assayed the ability of B7-1 to redistribute PSD95- and GluR1-positive (a subunit of AMPAR) puncta, which are normally localized to the postsynaptic spine head. To determine the effects of B7-1 on p75^NTR^-expressing neurons (Figure 4, A, B, E, and G), we took advantage of the cross-species reactivity between hB7-1 and mouse p75^NTR^ (Figure 1D) and added recombinant human B7-1–Fc (hB7-1–Fc) (750 nM, to ensure saturation of the p75^NTR^ receptor; see Supplemental Figure 6), human B7-2–Fc (hB7-2–Fc) (750 nM), or proNGF (10 nM) to cultured primary DIV18 hippocampal mouse neurons for 2 hours. Cells were fixed and actin localization, PSD95, GluR1, MAP2 (to distinguish dendrites from axons), and p75^NTR^ expression were evaluated using immunofluorescence (IF) microscopy (Figure 4A and Supplemental Figure 7C). Within 2 hours, neurons treated with hB7-1–Fc or proNGF (as a positive control) exhibited a significantly lower density of PSD95 and GluR1-positive puncta compared with control or hB7-2–Fc treated neurons (Figure 4, A, B, E, and G). We posited that the relocalization of PSD95 and GluR1 from the spine head to the dendritic shaft would result in a concomitant reduction in apposed presynaptic sites, as determined by anti-bassoon antibody binding. Indeed, hB7-1–Fc treatment induced a decline (~50%) in bassoon puncta, indicating that hB7-1 disassembles spines and eliminates synapses (Figure 4, E and F).

To establish that hB7-1–mediated PSD95 relocalization is dependent on p75^NTR^ binding, we cultured hippocampal neurons from p75^NTR^-knockout (*p75^–/–^*) mice. We added hB7-1–Fc, hB7-2–Fc, or proNGF and analyzed the density of PSD95-positive puncta after 2 hours. We observed that in *p75^–/–^* neurons, neither hB7-1 nor proNGF altered the density of PSD95-positive puncta, indicating that hB7-1–mediated changes in PSD95 localization are dependent upon binding to and signaling through p75^NTR^ (Figure 4, C and D).

Previous work demonstrated that proneurotrophin- or variant BDNF prodomain–mediated p75^NTR^ signaling in neurons requires the interaction of the prodomain with the coreceptors sortilin or SorCS2 (20). However, no binding could be detected between cells expressing B7-1 and cells expressing sortilin, SorCS1, SorCS2, or SorCS3 (Supplemental Figure 1). The differences in the binding interfaces present in the B7-1:p75^NTR^ and neurotrophin:p75^NTR^ assemblies, the membrane-based presentation of B7-1 versus soluble neurotrophins, and the lack of detectable interactions between hB7-1 and sortilin family members distinguished the hB7-1:p75^NTR^ and neurotrophin:p75^NTR^ interactions.

### Mixed culture assay to evaluate B7-1:p75^NTR^ activity demonstrates disassembly of PSD95 puncta, MAP2 reorganization, and loss of Bassoon colocalization in cultured hippocampal neurons.

**Figure 5:**
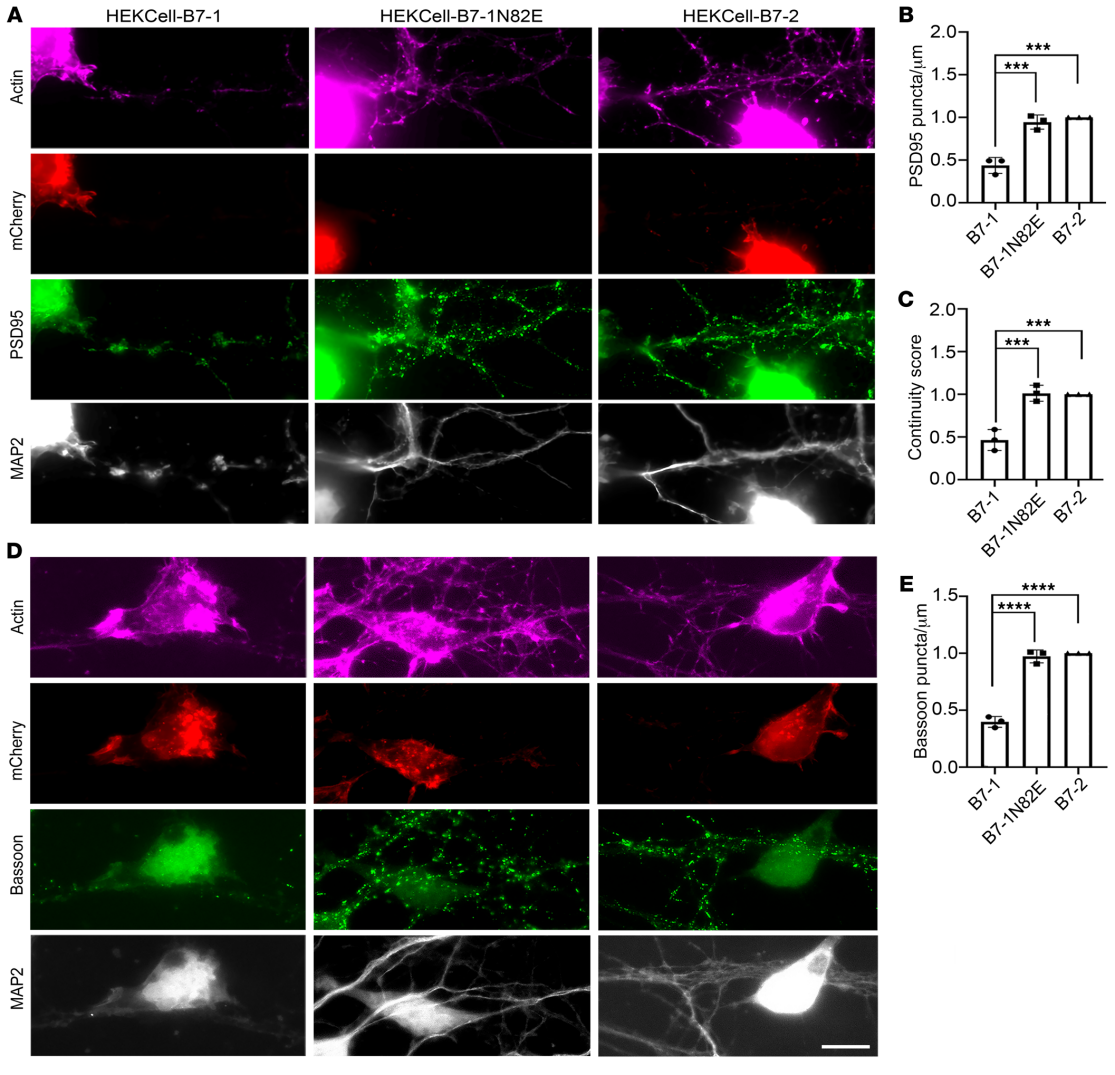
Cells expressing hB7-1 induce relocalization of PSD95 immunoreactivity, loss of opposing Bassoon puncta, and discontinuity of MAP2 in neurons. (**A**) Representative IF images of hippocampal neurons cocultured with HEK293–hB7-1, HEK293–hB7-2, or HEK293–hB7-1N82E. Differences in (**B**) PSD95 density and (**C**) MAP2 morphology in dendrites in direct contact with HEK-hB7 cell lines were quantified. (**D**) Representative IF images of hippocampal neurons cocultured with HEK293–hB7-1, HEK293–hB7-2, or HEK293–hB7-1N82E stained for bassoon, mCherry, MAP2, and actin. (**E**) Quantification of bassoon puncta density with indicated treatment. ***P < 0.001; ****P < 0.0001, 1-way ANOVA, Tukey’s multiple-comparisons test. *n* = 3 independent experiments. Experiments under each condition were performed in triplicate, and 13 to 15 dendritic segments were analyzed per condition. Error bars represent SEM. Scale bar: 20 μm.

B7-1:p75^NTR^ bioactivity was confirmed using an orthogonal method of B7-1 presentation. Because interactions between p75^NTR^ and B7-1 are likely to occur in *trans* between neurons and APCs, we developed a mixed culture assay system (33) that would more faithfully model the in vivo interaction. Stable HEK293 cell lines were generated, expressing either hB7-1, hB7-1N82E (which interacts with CD28, CTLA-4, and PD-L1, but has substantially reduced affinity for p75^NTR^; see Supplemental Figure 6), or hB7-2; these proteins were formatted as fusion proteins with intracellular mCherry (Supplemental Figure 8). To ensure similar levels of WT and mutant hB7-1 and hB7-2, cells expressing these fusion proteins were sorted and routinely evaluated by flow cytometry to ensure similar mCherry expression and appropriate ligand binding. To determine the effects of hB7-1–expressing cells on postsynaptic structures and apposing presynaptic input, stably transfected HEK293 cells were incubated with cultured DIV18 primary hippocampal mouse neurons for 4 hours and PSD95/bassoon density and MAP2 morphology were analyzed (Figure 5A). We observed that dendrites in direct contact with hB7-1–expressing cells displayed significantly lower PSD95 density and reduced density of bassoon from apposing presynaptic inputs, as compared with dendrites in direct contact with hB7-2– or hB7-1N82E–expressing cells (Figure 5, B and E). These results are in line with the phenotypic changes observed upon addition of recombinant hB7-1–Fc to WT hippocampal neurons (Figure 4).

Hippocampal neuronal dendrites in close proximity to cells expressing hB7-1N82E or hB7-2 exhibited uniform and continuous distribution of MAP2 throughout the dendrite (Figure 5, A and D). In contrast, dendrites of hippocampal neurons in proximity to hB7-1–expressing cells exhibited dystrophic morphology of MAP2-positive processes, with a distinct punctate MAP2-staining pattern (Figure 5A). To quantify the extent of microtubule degeneration induced by hB7-expressing cells, we developed a continuity score (see *MAP2 quantitation* in Methods) to determine the continuity of MAP2 along the length of neuronal processes. We evaluated dendritic processes in direct contact with hB7-1–, hB7-1N82E–, or hB7-2– expressing cells and observed that hB7-1–expressing cells significantly decreased the dendritic MAP2 continuity score compared with B7-2– or N82E-expressing cells (Figure 5C).

### Abatacept prevents dendritic damage induced by hB7-1:p75^NTR^.

**Figure 6:**
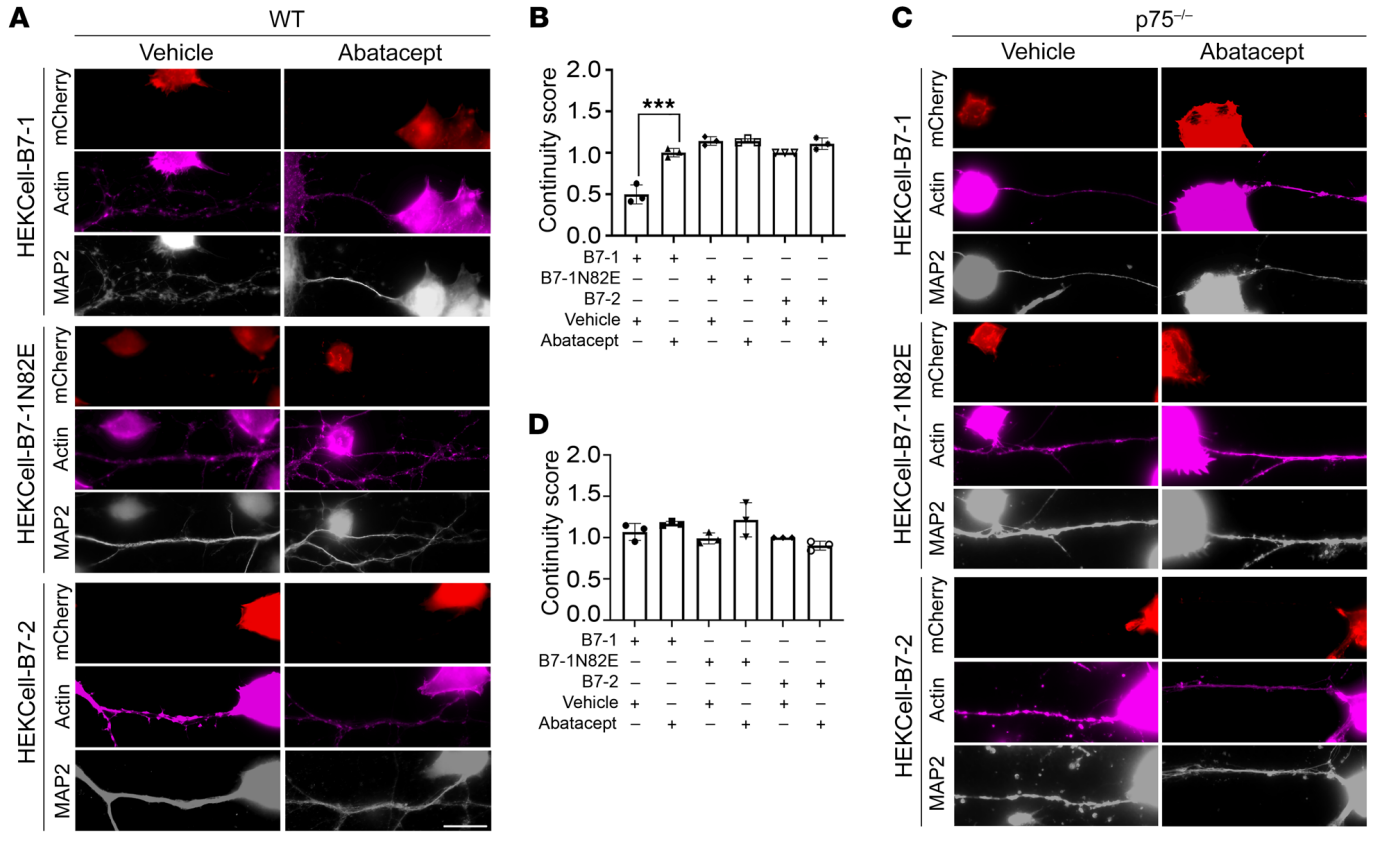
Cells expressing hB7-1 induce MAP2 discontinuity in WT but not *p75^–/–^* neurons, which can be inhibited by abatacept. (**A**) Representative IF images of WT hippocampal neurons cocultured with HEK293–hB7-1, HEK293–hB7-2, or HEK293–hB7–1N82E treated with or without 375 nM abatacept. Differences in (**B**) MAP2 morphology in dendrites in direct contact with HEK-hB7 cell lines were quantified. Treatment with 375 nM abatacept could prevent the MAP2-positive process degeneration induced by the HEK293–B7-1 cell line. ***P < 0.001, 2-way ANOVA. *n* = 3 independent experiments in which 12 to 14 individual neurons were analyzed per treatment per experiment. Scale bar: 20 μm. (**C**) Representative IF images of *p75^–/–^* hippocampal neurons cocultured with HEK293–B7-1, HEK293–B7-2, or HEK293–B7-1N82E treated with or without 375 nM abatacept. (**D**) No differences in MAP2 morphology in dendrites in direct contact with HEK-B7 cell lines (HEK293–B7-1, HEK293–B7-2, or HEK293–B7-1N82E treated with or without 375 nM abatacept) were found. *P* = 0.1860, 2-way ANOVA. *n* = 3 independent experiments. *P* < 0.01, 2-way ANOVA with multiple comparisons (WT coculture). Scale bar: 20 μm. See also Supplemental Figure 9. *n* = 3 independent experiments in which 12 to 14 individual neurons were analyzed per treatment per experiment. Error bars represent SEM.

We next investigated whether blockade of the hB7-1:p75^NTR^ interaction could impair the associated fragmentation of MAP2-positive processes. As soluble CTLA-4 fusion protein competed with p75^NTR^ for binding to hB7-1 (Figure 2D), we examined whether abatacept, an FDA-approved drug for the treatment of rheumatoid arthritis composed of the extracellular domain of human CTLA-4 and the Fc domain of hIgG1 (34), could interfere with the biological actions of hB7-1 on neurons. We confirmed that abatacept impaired hB7-1 binding to p75^NTR^ in a heterologous cell–based assay and that abatacept was nontoxic to mature hippocampal neuron cultures (Supplemental Figure 9). Addition of abatacept (375 nM) to hippocampal neurons cocultured with either hB7-1–, hB7-2–, or hB7-1N82E–expressing HEK293 cells was performed, and the continuity of MAP2-positive processes was quantified. Abatacept inhibited the degeneration of MAP2-positive processes induced by hB7-1–expressing cells (Figure 6, A and B, and Supplemental Figure 9). To corroborate that hB7-1–induced changes in neuronal morphology are p75^NTR^ dependent, hB7-1–, hB7-2–, and hB7-1N82E–expressing cells were cocultured with *p75^–/–^* hippocampal neurons. In this context, hB7-1–expressing cells did not elicit the dystrophic morphology and discontinuity of MAP2 processes, reinforcing that this effect is p75^NTR^ dependent (Figure 6, C and D). Furthermore, when abatacept was added to *p75^–/–^* neurons cultured alone or to neurons cocultured with hB7-1–expressing cells, no changes in MAP2 morphology were observed (Figure 6, A–D, and Supplemental Figure 9).

### Activated THP-1 cells express hB7-1 and induce MAP2 reorganization of hippocampal neurons.

**Figure 7:**
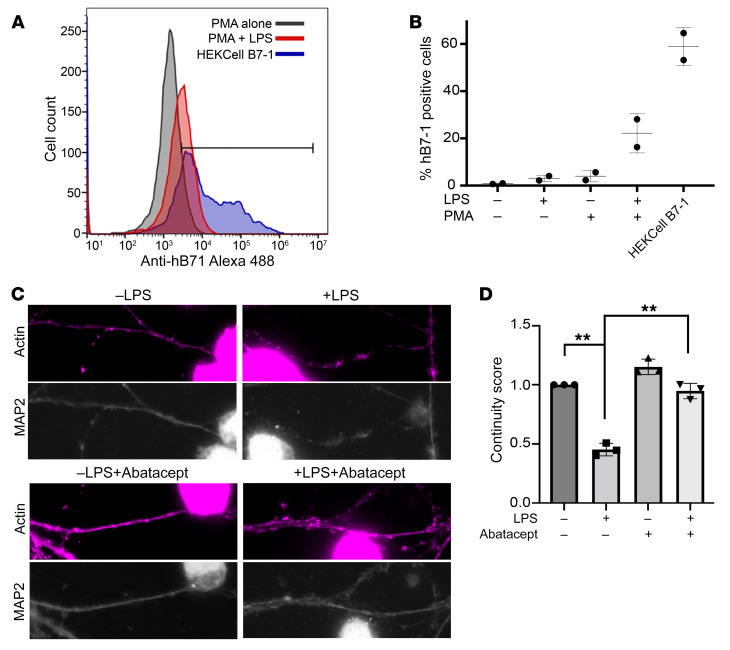
THP-1 cells expressing hB7-1 induce MAP2 degradation in neurons, which is inhibited by abatacept. (**A**) Cells were harvested at 24 hours after stimulation and stained for hB7-1 using an Alexa Fluor 488–conjugated anti-human B7-1 antibody. Antibody-stained THP-1 cells were analyzed by flow cytometry. Representative FACS plot of PMA-treated THP-1 cells (gray), THP-1 cells treated with both PMA and LPS (red), and HEK293 cells stably overexpressing hB7-1 (blue). (**B**) Quantification of *n* = 2 flow cytometry experiments. (**C**) Representative IF images of hippocampal neurons cocultured with Thp1 cells treated with PMA with or without LPS (1 μg/ml) with or without abatacept (375 nM). Original magnification, 43.7 × 16.09. (**D**) Differences in MAP2 morphology in dendrites in direct contact with THP-1 were quantified. **P < 0.001, 1-way ANOVA, Tukey’s multiple-comparisons test. *n* = 3 independent experiments. Each treatment was performed in triplicate, and 10 to 15 dendrites were analyzed for each condition. Error bars represent SEM.

Tissue-resident macrophages and microglia present antigens on MHC class I and class II molecules and express many different critical immunomodulatory ligands, including B7-1, which together act to regulate T cell activity. In healthy tissues, these APC populations act to maintain immune tolerance and homeostasis, but become activated in response to infection or inflammation (35, 36). To assess whether hB7-1 expressed endogenously in monocytic-derived macrophages is sufficient to induce similar B7-1–dependent changes in dendritic structure, we utilized THP-1 cells, a human monocytic cell line that can be differentiated into macrophage-like cells following exposure to phorbol myristate acetate (PMA) (37). Treatment of THP-1 cells with PMA for 24 hours causes a significant morphological change in which the cells become more adherent and polarized, express common macrophage markers (i.e., CD11b, CD14, etc.), respond to activation by LPS, and upregulate expression of key immunomodulatory ligands, including B7-1 and B7-2 (38). A similar LPS-dependent increase in B7-1 (CD80) expression was also previously observed for microglia cells, supporting the use of THP-1 cells treated with PMA and LPS to present hB7-1 in a manner more consistent with that of microglia cells in vivo (39). We demonstrated that THP-1 cells, which differentiate into macrophage-like cells in the presence of PMA, increased cell surface expression of hB7-1 following LPS activation (Figure 7, A and B). We then evaluated whether PMA+LPS-treated THP-1 cells induce reorganization of MAP2 in hippocampal neurons upon coculture. Using the MAP2 continuity score, we assessed MAP2-positive dendritic processes adjacent to PMA-treated THP-1 cells treated with or without LPS. THP-1 cells treated with PMA+LPS significantly decreased the dendritic MAP2 continuity score of adjacent hippocampal neurons compared with the effects of PMA-treated THP-1 cells not exposed to LPS (Figure 7, C and D). To confirm that this behavior reflected the effects of hB7-1, abatacept was added to parallel cultures, which resulted in an inhibition of degeneration of MAP2-positive processes induced by THP-1 cells (Figure 7, C and D). Taken together, these data demonstrate that B7-1:p75^NTR^ engagement is directly responsible for phenotypic changes in dendritic structure and that a drug in clinical use can impair the binding of B7-1 to p75^NTR^ and block dendritic damage.

### Delivery of hB7-1 into the subiculum, a hippocampal subregion, induces rapid pruning of dendritic spines in a p75^NTR^-dependent manner.

**Figure 8:**
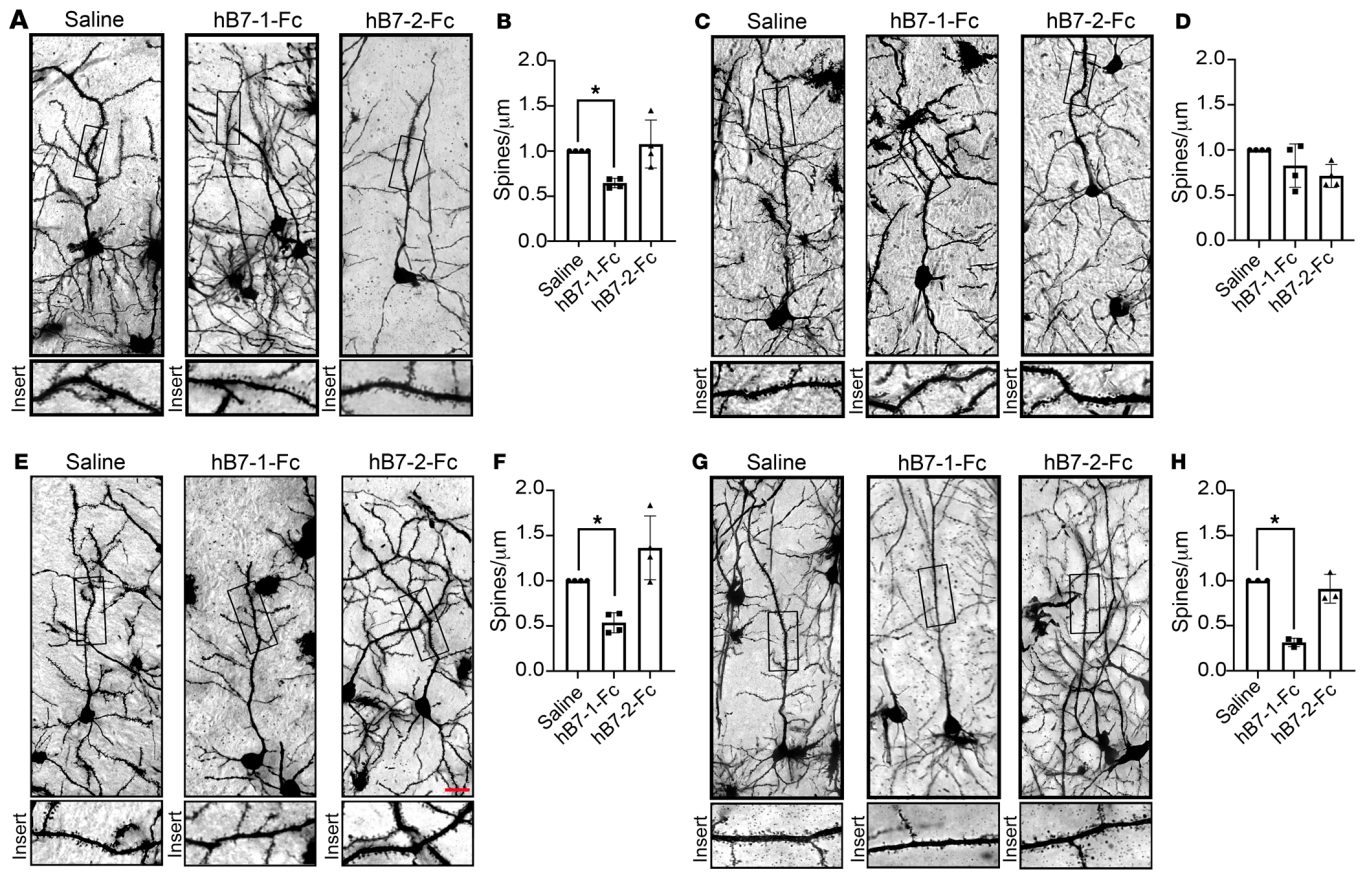
hB7-1–Fc injection into adult dSubiculum reduces total spine density. (**A**) Golgi-stained WT pyramidal neurons 3 hours after in vivo injection of hB7-1–Fc (100 ng), hB7-2–Fc (100 ng), or saline into the dSubiculum area of the hippocampal formation at P75. (**B**) There was a significant reduction in total spine density after 3 hours of hB7-1, compared with saline and the hB7-2 group. *n* = 4 mice/condition. (**C**) Golgi-stained *p75^–/–^* pyramidal neurons 3 hours after in vivo injection of hB7-1–Fc (100 ng), hB7-2–Fc (100 ng), or saline into the dSubiculum area of the hippocampal formation at P75. *n* = 4 mice/condition (**D**) There were no significant changes in total spine density after 3 hours for the hB7-1 or hB7-2 group compared with the saline group. *n* = 4 mice/condition. (**E**) Golgi-stained WT pyramidal neurons 3 hours after in vivo injection of hB7-1–Fc (200 ng), hB7-2–Fc (200 ng), or saline into the dSubiculum area of the hippocampal formation at P75. (**F**) There was a significant reduction in total spine density after 3 hours of hB7-1, compared with the saline and hB7-2 groups. *n* = 4 mice/condition. (**G**) Golgi-stained WT pyramidal neurons 24 hours after in vivo injection of hB7-1–Fc (200 ng), hB7-2–Fc (200 ng), or saline into the dSubiculum area of the hippocampal formation at P75. (**H**) There was a significant reduction in total spine density after 24 hours of hB7-1, compared with the saline and hB7-2 groups. *n* = 4 mice per condition. The apical dendrite segment 50 to 150 μm away from the cell soma was chosen for quantification. *P < 0.05, 1-way ANOVA, post hoc Tukey’s test. Scale bar: 20 μm. 15–20 neurons/brain. Data are represented as mean ± SEM. Error bars represent SEM. See also Supplemental Figure 10.

The subiculum is a region of the hippocampal formation that is critically involved in memory formation and retrieval (40) and expresses p75^NTR^ during adulthood (Supplemental Figure 10). Furthermore, atrophy and degeneration of subicular neurons is an indicator of the onset of AD (41). To determine whether hB7-1 can affect synaptic morphology in neurons of the dorsal subiculum (dSubiculum), we developed a model system in which soluble hB7-1 is delivered to the hippocampus via direct injection (Supplemental Figure 10). Three hours following a single dose of hB7-1–Fc or hB7-2–Fc (100 ng or 200 ng) or diluent control (Supplemental Figure 10) delivered to the dSubiculum, brains were harvested and changes in dendritic spine density evaluated using Golgi staining (42). In WT mice expressing p75^NTR^, 100 ng hB7-1–Fc delivery resulted in a 40% reduction in spine density, whereas delivery of hB7-2–Fc had no effect (Figure 8, A and B). In parallel experiments using *p75^–/–^* mice, neither acute injection of hB7-1–Fc nor of hB7-2–Fc (100 ng) led to dendritic spine pruning compared with effects in *p75^–/–^* mice injected with diluent (Figure 8, C and D). Furthermore, hB7-1 (200 ng) led to greater than 50% reduction in spine density, where equivalent hB7-2 delivery again had no effect (Figure 8, E and F). Finally, we observed the effects of hB7-1 (200 ng) on dendritic morphology 24 hours after injection, when hB7-1–Fc and hB7-2–Fc could no longer be detected in the subiculum (Supplemental Figure 10). While no effect was seen 24 hours after hB7-2–Fc injection, there was a greater than 60% loss in dendritic spine density 24 hours after hB7-1 injection (Figure 8, G and H). These data demonstrate that hB7-1–mediated changes in dendritic spines observed upon hB7-1–Fc injection into the brain are dependent on p75^NTR^ and that these changes can persist once B7-1 is no longer present. Furthermore, these experiments demonstrate that hB7-1:p75^NTR^ signaling can occur in vivo.

## Discussion

Through an unbiased screen for binding partners of the immunomodulatory ligand hB7-1, we identified an interaction with p75^NTR^, a cell surface receptor previously established to regulate a variety of neuronal functions through binding to neurotrophin ligands. To our knowledge, the interaction between p75^NTR^ and hB7-1 represents the first known instance of an interaction between a neurotrophin receptor and an immunological ligand. We confirmed the direct hB7-1:p75^NTR^ interaction with 3 distinct biochemical assays and defined the molecular determinants through mutational analysis. A model based on the existing crystal structures of hB7-1 and p75^NTR^, imposing close proximity between p75^NTR^F136 and hB7-1N82 (Figure 2), would seem to preclude an antiparallel binding interaction because of clashes between the IgC domain of hB7-1 and the CRD1 and CRD2 domains of p75^NTR^. Therefore, it is possible that hB7-1:p75^NTR^ binding requires a structural rearrangement or that hB7-1 and p75^NTR^ bind via an orthogonal pose similar to CTLA-4:B7-1, which is typical of many receptor/ligand interactions within the immunoglobin superfamily.

The promiscuous binding of primate B7-1 orthologues with p75^NTR^ orthologues from multiple mammalian species, coupled with the apparent restriction of this interaction to primates, suggests that p75^NTR^ binding is a property that has been recently acquired by alterations in the primate B7-1 gene rather than alteration of the primate p75^NTR^ gene. However, a more thorough phylogenetic analysis will be required to identify the evolutionary origin of this gain of function in primate B7-1 orthologues. Notably, amino acid differences in human and nonhuman primate B7-1, as compared with B7-1 of lower mammals, map to the regions of B7-1 that mediate p75^NTR^ binding (Supplemental Figure 2A). Because of these characteristics, existing rodent models will fail to recapitulate aspects of human pathophysiology associated with B7-1–mediated p75^NTR^-dependent processes in a variety of pathological states, including EAE (21), acute traumatic injury (43), AD (15), and cerebral ischemia (44), which are all conditions characterized by p75^NTR^ induction and by inflammatory changes, including accumulation of APCs.

While we demonstrate that hB7-1:p75^NTR^ engagement may negatively affect neuronal synapses, it is well recognized that APCs, such as microglia, regulate neuronal structure and function, often in the context of inflammatory stimuli or in neurodegenerative conditions. Imaging studies demonstrate that microglia dramatically alter their morphology and extend processes to survey the surrounding environment in the setting of injury or an inflammatory challenge (45), with microglial processes projecting to neuronal synapses to monitor and regulate synaptic activity (46, 47). The ligand/receptor complexes that regulate microglial/neuronal interactions are incompletely understood; however, several candidate systems have been identified, including neuronally expressed proteins CD200 and fractalkine (48) and CD200 receptor (CD200R), expressed on macrophages and microglia. CD200/CD200R signaling maintains a resting or nonactivated microglial state (49, 50), and defects in CD200 signaling, which result in increased microglial activation, are associated with neuroinflammatory conditions such as MS and AD. Impaired CD200 signaling is also associated with an increased aging phenotype in the brain (51, 52). Microglia utilize complement proteins C1q and C3 to mediate synapse elimination. In mouse models of AD, C3 and C1q associate with synapses, and microglia recognize and engulf these synapses in a complement receptor 3–dependent (CR3–dependent) manner (53). While it is established that C1q can “tag” synapses for degradation in order to mediate proper synaptic pruning during development (54), the mechanisms utilized in neurodegenerative models are incompletely characterized.

Similarly to the complement proteins, which are expressed by activated APCs under inflammatory conditions, the p75^NTR^ receptor is an established component of the inflammatory response in the brain. In response to inflammatory insults or acute injury, such as traumatic brain or spinal cord injury (55, 56), viral infection (57), and stroke (44), and in the setting of complex neurological disorders, including AD (58) and EAE, (the mouse model of MS) (15, 21), p75^NTR^ is upregulated and can adversely affect neuronal function. Unsurprisingly, the costimulatory molecule B7-1, a ligand for p75^NTR^, is associated with many of these same diseases (59). Expression of this molecule is documented on activated microglia and brain-infiltrating macrophages in response to exposure to LPS and proinflammatory cytokines (3, 4), traumatic brain injury (3, 60), infections of the brain (5, 61), and neurodegenerative diseases such as MS and AD (6–8, 62).

While C3- and C1q-mediated synaptic loss can be readily studied in mice, murine B7-1 fails to activate murine p75^NTR^. Thus, effects of human B7-1:p75^NTR^ engagement represent a previously unappreciated mechanism by which brain-infiltrating APCs or activated microglia may regulate neuronal structure and function. These studies further suggest that blockade of hB7-1:p75^NTR^ interaction represents a potential therapeutic strategy for inflammatory neuropathologies and neurodegenerative conditions such as MS, AD, and similar pathologies. While our results suggest that abatacept can block hB7-1:p75^NTR^–mediated cellular processes in vitro, additional studies will be required to evaluate in vivo efficacy.

It should be noted that in addition to the nervous system, p75^NTR^ expression is also observed in numerous tissues, including the musculature (63), immune system (64, 65), and cardiovascular system (66), and in non–nervous system diseases, such as chronic arthritis (67), diabetes (68), and cancer (69), which may be affected by B7-1 binding. Collectively, this work provides biochemical and functional data for continued dissection of hB7-1:p75^NTR^ interaction and the potential development of therapeutic strategies for a variety of human diseases.

## Methods

### Tissue culture and transient transfection.

HEK293 suspension cells were cultured according to the manufacturer’s instructions and transfected with PEI as previously described (24). See Supplemental Methods for details.

### B7-1 and p75^NTR^ site-directed mutagenesis.

All site-directed mutagenesis of B7-1 and P75^NTR^ was performed using high-fidelity KOD Hot State polymerase, 2 mM dNTPs, and 4 mM MgCl_2_ (EMD Millipore, 71086-3). The template used for the p75 mutagenesis included the coding sequence for full-length human p75^NTR^ cloned between the XHOI and ECORI sites of the Clontech N1 GFP vector. For B7-1, the template used included the full-length native human B7-1 coding sequence cloned between the XHOI and ECORI sites of the Clontech N1 mCherry vector by In-Fusion (Clontech). Mutagenesis was performed as previously described with modifications (24). See Supplemental Methods for details.

### Cell-cell binding experiments.

Three mammalian expression display libraries were screened: a library of 479 combined Ig superfamily and TNFRSF constructs tagged with cytosolic GFP, a library of B7-1 point mutations tagged with cytosolic mCherry, and a library of p75^NTR^ point mutations tagged with cytosolic mCherry. Query ligands were tagged with the opposite color cytosolic tag (mCherry for screening against the Ig and TNFR SFs library, and the p75^NTR^ point mutant library, GFP for screening against the B7-1 point mutant library). For screening, libraries and query ligands were transfected in small scale as described above. Two days after transfection, cells were diluted to 1 × 10^6^ cells/mL in PBS 0.2% BSA, pH 7.4. Binding reactions were set up in 96-well V-bottom plates by mixing equal volumes of challenger (scFv-expressing cells) and library-expressing cells. After binding, cell-cell conjugates were analyzed by flow cytometry using a HyperCyt ample loader coupled to a BD Accuri flow cytometer or a SONY Spectral Analyzer (SA3800). The percentage bound was calculated as the number of double-positive events (GFP and mCherry) divided by the total number of cells. For mutagenesis studies, percentage binding was calculated as the percentage bound of a given mutant divided by the percentage bound of the WT interaction.

### Purification of recombinant Fc-fusion protein.

To clone B7-1, CTLA-4, p75^NTR^, and PD-L1 Fc-fusion proteins, full-length WT or mutant ectodomains (B7-1: residues 35–233) were subcloned into a LIC vector containing a C-terminal deca his-tagged Fc domain (mIgG2a-His10 or hIgG1-His10). All expression constructs as well as mIgG2a and hIgG1 isotype control constructs were transiently expressed in 50 mL of Expi293 (Thermo Fisher Scientific) suspension cells and transfected according to manufacturer guidelines. Protein was purified as previously described (24). See Supplemental Methods for details.

### Microbead cell flow cytometry–binding assay.

Microbead cell flow cytometry binding assays were performed as previously described with modifications (24). See Supplemental Methods for details.

### Recombinant protein/bead titration experiments.

For recombinant protein/bead titration experiments, hB7-1 (residues 35–233) was subcloned into a LIC vector containing a C-terminal 10X His and AVI-tagged hIgG1 domain hIgG1-His10 and transiently expressed in Expi293 cells stably expressing a copy of the BirA gene, which enzymatically attaches a molecule of biotin to the AVI tag and was purified as described above. Biotinylation was confirmed by streptavidin pulldown and SDS-PAGE. 15 μg of B7-1–Fc biotin was incubated with 5 μL of streptavidin-coated beads (Bangs Laboratories, CP01003) in a total volume of 500 μL PBS, 0.2% BSA, pH 7.4 at room temperature for 60 minutes. Beads were washed by pelleting at 1,000*g* for 5 minutes, and B7-1 loading was confirmed using an anti–B7-1 antibody (RND Systems, catalog AF140) and flow cytometry. CTLA-4/mIgG2a, p75^NTR^-mIgG2a, and mIgG2a control were titrated onto 5 μL of B7-1–loaded beads in a 96-well plate at concentrations of 0 to 100 nM for 30 minutes, washed twice, and then incubated with an Alexa Fluor 647–labeled antibody (Thermo Fisher, A-21235) against mIgG2a. Binding was detected by flow cytometry on a SONY Spectral Analyzer (SA 3800). Binding curves were fit using the equation *Y* = B_max_ × *X^h^*/(*Kd^h^* + *X^h^*), where *Y* = relative fluorescence units, B_max_ = maximum possible ligand binding, *X*= concentration, *h* = hill coefficient, and *Kd* = dissociation constant.

### Competition experiments.

Competition experiments using ligand bound to protein A beads were performed as previously described (24) with modifications. See Supplemental Methods for details.

### Flow cytometry protein/cell titration assay.

Flow cytometry protein-binding experiments were performed as previously described (24) with modifications. See Supplemental Methods for details.

### Hippocampal neuronal cultures.

For neuronal cultures, C57BL/6N mice from Charles River Laboratories and *p75^–/–^ mice* (B6.129S4-Ngfrtm1Jae/J)^52^ from Jackson Laboratories were used. DIV18-21 primary hippocampal neurons were prepared from pregnant dams as previously described (4). The cells were maintained in culture for 18 to 21 days without any media changes/additions. Neurons were starved for 2 to 3 hours in neurobasal medium supplemented with 0.5% glucose. Following starvation, neurons were treated with 0.75 μM (40 ng/mL) of hB7-1–Fc and hB7-2–Fc proteins (Sino Biological; 10698-H03H and 10699-H03H) at 37°C/5% CO_2_. Two hours after protein application, neurons were briefly washed with prewarmed HBSS and fixed with prewarmed 4% paraformaldehyde/4% sucrose solution for 15 minutes at room temperature.

### Mixed coculture assay.

Mixed culture assays using HEK293 cells and cultured neurons were prepared as described (33) with modifications. HEK293 cells were washed with 10 mL warm neurobasal medium supplemented with B27, 1 mM sodium pyruvate, 6 mM Glutamax, 100 U/mLpenicillin-streptomycin, and 4 μM cytosine-1-b-d-arabinofuranoside per 100 × 20 cm culture dish to prevent HEK293 overgrowth. HEK293 cells were triturated without trypsinization, counted, and seeded at a density of 20 × 10^3^ per 12 mm glass coverslip with hippocampal neuronal cultures (WT or *p75^–/–^*). The mixed culture was incubated for 4 hours and fixed with prewarmed 4% paraformaldehyde/4% sucrose solution for 15 minutes at room temperature.

THP-1 cells (ATCC, TIB-202) were differentiated by adding 50 ng/mL PMA (Thermo, J63916.LB0) and incubated for 24 hours in a 37°C humidified CO_2_ incubator. After 24 hours, the media were replaced, and the cells were kept for another 24 hours. The media were replaced with fresh RPMI media with or without LPS at 1 mg/mL. The cells were incubated for another 24 hours. After 24 hours of stimulation, cells were washed 3 × 30 minutes and were detached by scraping, then counted and seeded at a density of 20 × 10^3^ per 12 mm glass coverslip with WT hippocampal neuronal cultures. The mixed culture was incubated for 4 hours and fixed with prewarmed 4% paraformaldehyde/4% sucrose solution for 15 minutes at room temperature. For flow cytometry, differentiated and stimulated THP-1 cells (100 K) were harvested in 1× PBS and 0.2% BSA and stained with 0.5 μg of Alexa Fluor 488 anti-human CD80 (B7-1) antibody (BioLegend, catalog 305214). Cells were gated for positive hB7-1 expression based on unstained THP-1 populations.

### Immunocytochemistry.

For immunocytochemistry of the hippocampal neuronal cultures and mixed culture assays, cells were permeabilized and blocked with 3% fetal bovine serum, 3% BSA, and 0.1% Triton X-100 in PBS for 15 minutes at room temperature. Primary antibodies were applied overnight at 4°C, and subtype-specific Alexa Fluor fluorescent secondary antibodies were added for 40 minutes at room temperature. Coverslips were mounted with ProLong Gold antifade reagent (Invitrogen; P36934). Primary antibodies were anti-PSD95 (Abcam; catalog ab2723), anti-MAP2 (Abcam; catalog ab92434), anti-bassoon (Enzo, ADI-VAM-PS003-D clone, catalog SAP7F407), anti-GluR1 (Invitrogen, catalog PA1-46151), and anti-p75^NTR^ (R&D Systems; catalog AF1157). Secondary antibodies were Alexa Fluor antibodies (Life Technologies), except for the 405 nm fluorescent DyLight (Jackson ImmunoResearch Laboratories; catalog 102649-302). For visualization of actin cytoskeleton, Alexa Fluor 546 and Alexa Fluor 647 phalloidin (Life Technologies, Thermo Fisher Scientific; A22283 and A30107) were used.

### Stereotactic injections.

Mice were maintained with a 12-hour light/12-hour dark cycle at 18°C to 22°C and had ad libitum access to water and food. All mice were healthy with no obvious behavioral phenotypes, and none of the experimental mice were immune compromised. C57BL/6 mice (2.5 months old) were used for stereotactic microinjection of hB7-1–Fc or hB7-2–Fc (100 ng; Sino Biological; 10698-H03H and 10699-H03H). Mice were anesthetized with a ketamine and xylazine cocktail (100 mg and 10 mg/mL) at dosages of 0.1 mL per 10 g of total body weight and then mounted on a stereotactic frame (David Kopf Instruments) prior to surgical exposure of the skull. Stereotactic coordination was performed by using an electrical drill mounted on a manipulator (David Kopf Instruments). The settings for targeting the dorsal subiculum (dSubiculum) were the following: AP = 3.52 mm, ML = 2.5 mm, DV = 1.75 mm. Microinjections were performed using a Nanoject II Auto-Nanoliter Injector (Drummond Scientific Company). A total of 200 nL of protein was injected into each animal. The needle was kept in place for an additional 5 minutes prior to withdrawal to minimize leakage up the needle track after the injection. Three hours later, mice were deeply anesthetized with sodium pentobarbital.

### Abatacept treatment.

For hippocampal neuron culture, abatacept (Bristol Myers Squibb) (70) or human IgG (R&D Systems, 110-HG-100) was added at a concentration of 375 nM, incubated for 4 hours, and fixed with prewarmed 4% paraformaldehyde/4% sucrose solution for 15 minutes at room temperature.

For WT or *p75^–/–^* hippocampal neuron and HEK293 cells mixed culture/Thp1 cells, abatacept was added at a concentration of 375 nM 10 minutes before seeding HEK293 cells, HEK293-B7–expressing cells, or THP-1 cells with or without LPS (see *Mixed coculture assay*), incubated for 4 hours, and fixed with prewarmed 4% paraformaldehyde/4% sucrose solution for 15 minutes at room temperature.

### Immunohistochemistry.

Three hours following stereotactic injection of hB7-1–Fc, hB7-2–Fc, or diluent control, mice were deeply anesthetized with sodium pentobarbital and perfused transcardially using the Perfusion Two Automated Pressure Perfusion system (Leica Microsystems) with 0.9% saline, followed by of 4% paraformaldehyde in 0.01M sodium phosphate buffer (PB) (pH 7.4) at a flowing rate of 25 mL/min. The brains were then removed and post-fixed with 4% paraformaldehyde in 0.01 M sodium PB at 4°C overnight and transferred to a sucrose solution (30% sucrose in 0.1M PB) at 4°C for 48 to 72 hours. Coronal sections (thickness, 40 μm) were prepared using a freezing microtome. The sections were set in an antifreeze solution (30% glycerol, 30% ethylene glycol, and 40% 0.25M PB) and stored at –20°C. Free-floating serial sections were washed (3 times for 10 minutes each) in PBS and incubated for 1 hour in a blocking solution containing 5% normal donkey serum and 1% BSA in PBS with 0.08% Triton X-100 (PBS–Tx), following incubation in primary antibodies (p75^NTR^, NeuN, MilliporeSigma, catalog ABN78) diluted in the blocking solution mentioned above and incubated 24 to 48 hours at 4°C with rotating. After washing in PBS, sections were incubated for 2 hours with secondary antibodies (anti-rabbit, Fisher Scientific, catalog A-21206; anti-human IgG, Thermo Fisher, catalog SA5-10128, conjugated with DyLight 594) and washed. The sections were mounted and air-dried overnight in the dark. Slides were coverslipped by water-soluble glycerol-based mounting medium containing DAPI and sealed with nail polish.

### Golgi staining.

Three hours following stereotactic injection of hB7-1–Fc, hB7-2–Fc, or diluent control, mice were euthanized using Euthasol (Virbac, 0.1 mL/10 g body weight). The brains were rapidly removed and were processed following a modified Golgi-Cox protocol, as previously described (71). See Supplemental Methods for details.

### Brightfield and fluorescence microscopy.

Recombinant protein assay images were acquired on an inverted fluorescent Nikon Eclipse Ti2-E microscope equipped with an Andor Zyla 5.5 sCMOS camera and a Lumencor SOLA-SE II light engine. The objectives used were CFI Nikon PlanApo Lambda ×20 with 0.75 NA and CFI Nikon Plan Apo Lambda ×60 oil lenses with 1.40 NA and a ×1.5 adapter. Acquisition settings were kept consistent across all conditions within a given experiment. For high magnification, mixed culture assay images were taken on a Zeiss Cell Observer SD confocal microscope with a Yokagawa CSU-X1 spinning disk, Plan-Apochromat ×63/1.4 M27 objective paired with a ×1.2 adapter to a Photometrics Evolve 512 EMCCD camera that was used for image acquisition. The laser lines used were 405 nm, 488 nm, 561 nm, and 639nm.

### MAP2 quantitation.

For quantitative assessment of MAP2 integrity, the MAP2 channel alone was used and processed with ImageJ software (NIH). Background was subtracted and a threshold was applied. Secondary and tertiary dendritic branches were traced with a linear path using the actin channel. The plot profile tool was then used to quantify the thresholded MAP2 signal along the dendrite. The area under the curve of each linear path was then divided by 255 multiplied by path length and multiplied by 100 to determine percentage of continuity of MAP2 signal through the dendrite. The code used to analyze MAP2 continuity is provided in the Supplemental Methods.

### Statistics.

For quantitative assessment of synaptic puncta, the PSD95/ bassoon/GluR1 channels were analyzed and processed with FIJI software (ImageJ 2.0.0-rc-43/1.52n). Secondary and tertiary dendritic branches were selected blind, based on the actin and MAP2 channels. Background was subtracted and a threshold was applied. PSD95/bassoon/GluR1 puncta on secondary and tertiary dendritic branches were counted. Finally, the length of the branch was measured to assess the density. Experiments were performed with at least triplicate replicates per condition and independently replicated 3 times with similar results. All data were acquired and analyzed in a blinded fashion, and experiments were repeated by different individuals to ensure reproducibility. All data were analyzed with GraphPad Prism 5.0 software. Experimental data were statistically analyzed by 1-way ANOVA with Bonferroni’s post hoc tests to control for multiple comparisons (for 3 or more group comparisons) or 2-way ANOVA with Bonferroni’s post hoc tests (to assess statistical significance between means), as indicated within individual figure legends. All statistical parameters are presented as mean ± SEM. Additional statistical information is provided in Supplemental Methods.

### Study approval.

All of the animal experiments were conducted in accordance with the *Guide for the Care and Use of Laboratory Animals* (National Academic Press, 2011) and approved by the Institutional Animal Care and Use Committee at Weill Cornell Medical of Cornell University.

## Author contributions

NCM, SCGT, FSL, BLH, and SCA conceived the project and designed the experiments. NCM performed the biochemical experiments and analyzed the data. RSS, VD, and MKL performed the neuronal culture experiments. RS and VD acquired the coculture experiment images. VD performed the in vivo injections, and VD and RS analyzed the in vivo experiments. NCM, RSS, VD, MKL, FSL, and BLH analyzed the data from neuronal culture experiments. UP, under supervision of NCM, performed the PTPRF:p75^NTR^ screening experiment. NCM and SMO developed the MAP2 analysis. NCM, NGH and AC generated stable cell lines. NGH generated plasmids encoding the mouse homologues of CD28 and CTLA-4 and assisted with biochemical experiments. CS assisted with HEK293F cell culture. SCGT designed the immunoglobulin superfamily (IGSF) and TNFRSF libraries and screening methods. SCGT and CS conducted high-throughput screening. NCM, RSS, SJG, SCGT, FSL, BLH, and SCA interpreted data. SJG, SCGT, FSL, BLH, and SCA supervised research. FSL, BLH, and SCA provided funding. NCM generated the initial manuscript. NCM, VD, SCGT, FSL, BLH, and SCA generated the revised manuscript with comments from SJG. All authors approved the final manuscript.

## Supplementary Material

Supplemental data

Supplemental table 1

Supplemental table 2

Supplemental table 3

## Figures and Tables

**Table 1:**
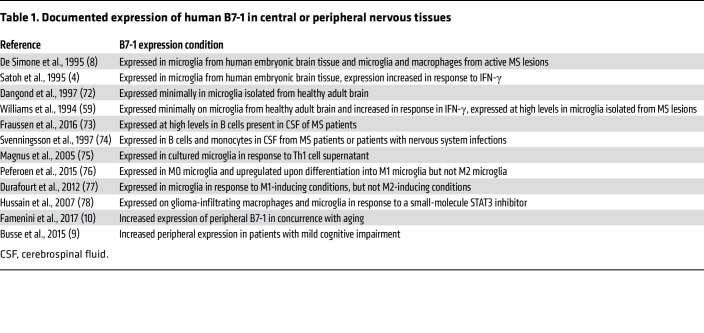
Documented expression of human B7-1 in central or peripheral nervous tissues

## Version 1 09/15/2022

In-Press Preview

## Version 2 11/15/2022

Electronic publication
