# Supplementary material for: Human immunomodulatory ligand B7-1 mediates synaptic remodeling via the p75 neurotrophin receptor
Source: J Clin Invest. 2022 Nov 15;132(22):e157002. doi: 10.1172/JCI157002 (PMC9663165; doi:10.1172/JCI157002)
Supplement: Supplemental data [file jci-132-157002-s110.pdf]

Extended Data for:

Human immunomodulatory ligand B7-1 mediates synaptic remodeling via the p75 neurotrophin receptor

Nicholas C. Morano[1,2,+], Roshelle S. Smith[3,+], Victor Danelon[3,+], Ryan Schreiner[4], Uttsav Patel[1], Natalia G. Herrera[1], Carla Smith[1], Steven M. Olson[5], Michelle K. Laerke[3], Alev Celikgil[1], Scott J. Garforth[1], Sarah C. Garret-Thomson[1], Francis S. Lee[6,], Barbara L. Hempstead[3,*], Steven C. Almo[1,*]*

[+]Contributed equally

*Corresponding Authors

[1]Department of Biochemistry, Albert Einstein College of Medicine

[2]Zuckerman Mind Brain Behavior Institute, Columbia University

[3]Department of Medicine, Weill Cornell Graduate School of Medical Sciences

[4]Division of Regenerative Medicine, Hartman Institute for Therapeutic Organ Regeneration, Ansary Stem Cell Institute, Department of Medicine, Weill Cornell Medicine

[5]Department of Computer Science, Georgia Institute of Technology

[6]Department of Psychiatry, Weill Cornell Medicine

Correspondence and requests for materials should be addressed to Steven C. Almo, Albert Einstein College of Medicine, 1300 Morris Park Ave, Bronx NY, 10461, steve.almo@einsteinmed.edu, 718-430-2046, Barbara L. Hempstead, Department of Medicine, Weill Cornell Graduate School of Medical Sciences, 1300 York Ave, New York, NY, blhempst@med.cornell.edu, 212-746-6215, or Francis S. Lee, Department of Psychiatry, Weill Cornell Medicine, 1300 York Ave, New York, NY, fslee@med.cornell.edu, 212-746-5403.

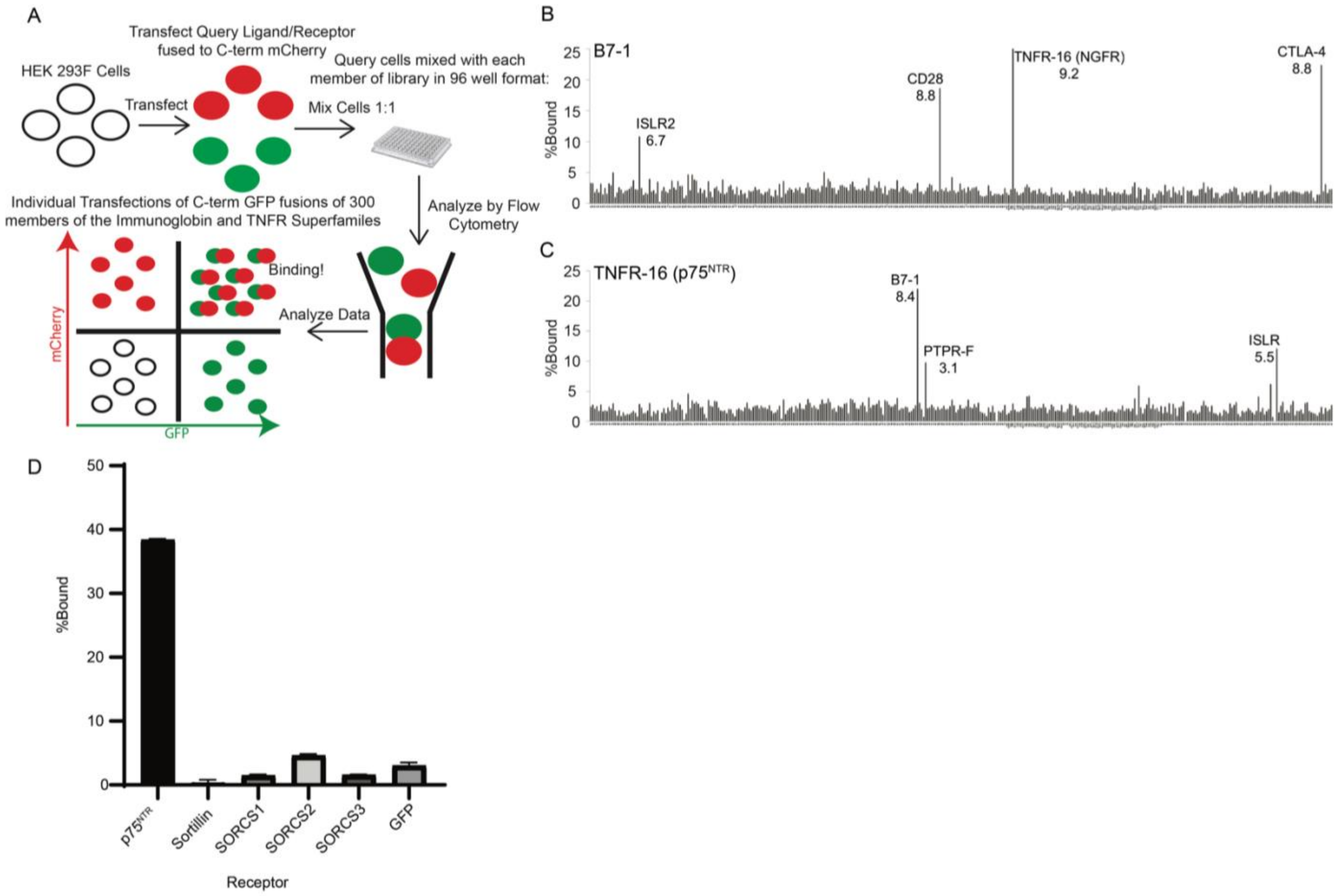

Figure S1: High-throughput Screening Identifies Uncharacterized p75^{NTR} Interactions: A) Schematic of high-throughput cell:cell screening platform **B)** Screening B7-1 against 300 members of the IG and TNFRSF identified interactions with CD28, CTLA-4, and p75^{NTR}, B7-1:ISLR2 interaction was not reproducible **C)** Screening p75^{NTR} against the same library identified interactions with B7-1 and PTPRF, p75^{NTR}:ISLR interaction was not reproducible. See supplemental table S1 **D)** HEK293F cells were transfected with B7-1-mCherry and screened against cells expressing either p75^{NTR}-GFP, Sortilin-GFP, SORCS1-GFP, SORCS2-GFP, SORCS3-GFP, or GFP Control. Binding could only be detected to cells expressing p75^{NTR} cells, n = 3 independent experiments, each with 1 biological replicate.

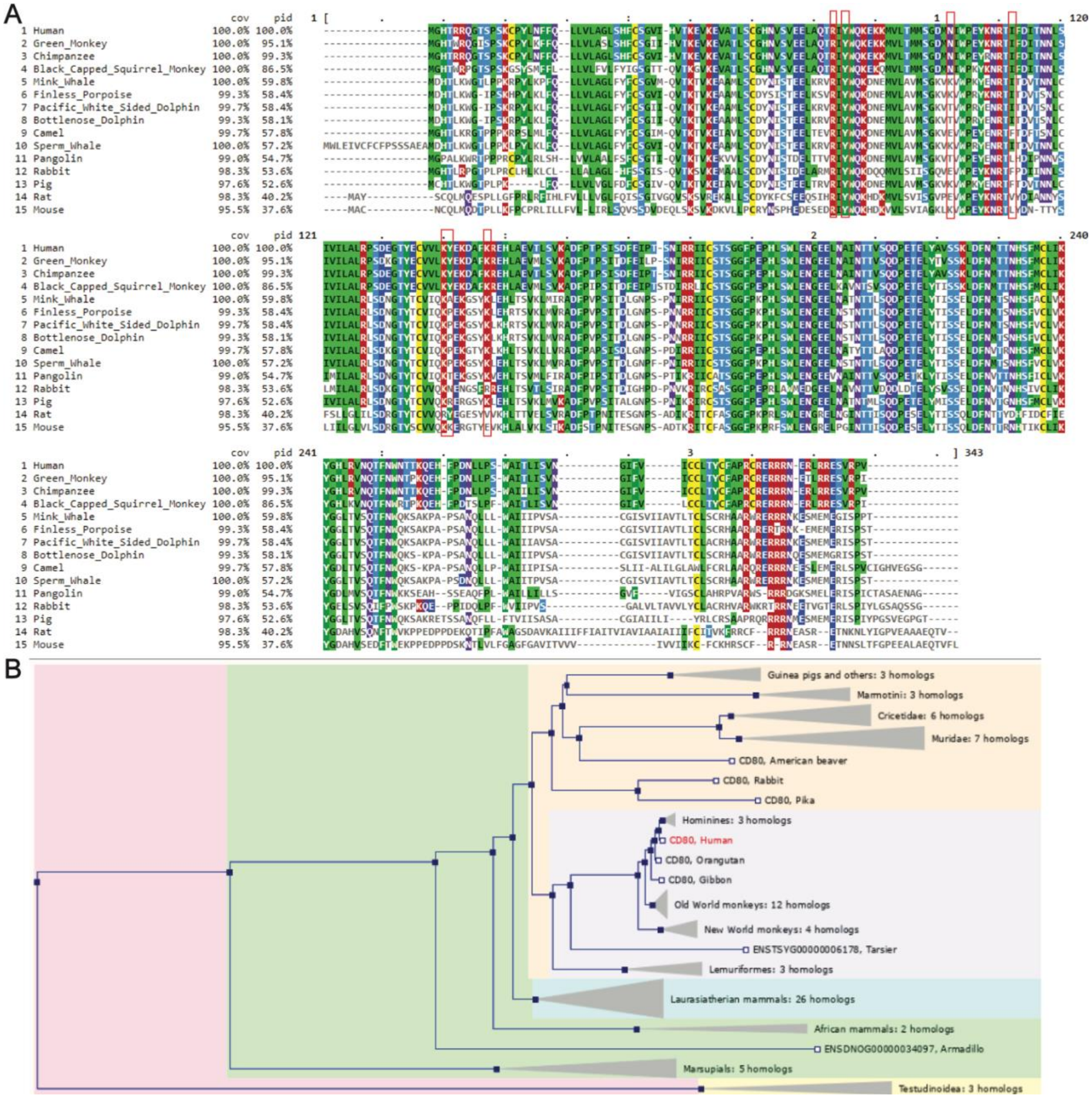

Figure S2: B7-1 Homology: A) Clustal Omega analysis of human B7-1 aligned with homologues from 14 mammalian species, in order of % identity, regions of importance for p75^{NTR} binding are indicated by a red box **B)** Phylogenetic analysis of the B7-1 gene generated using Ensembl.

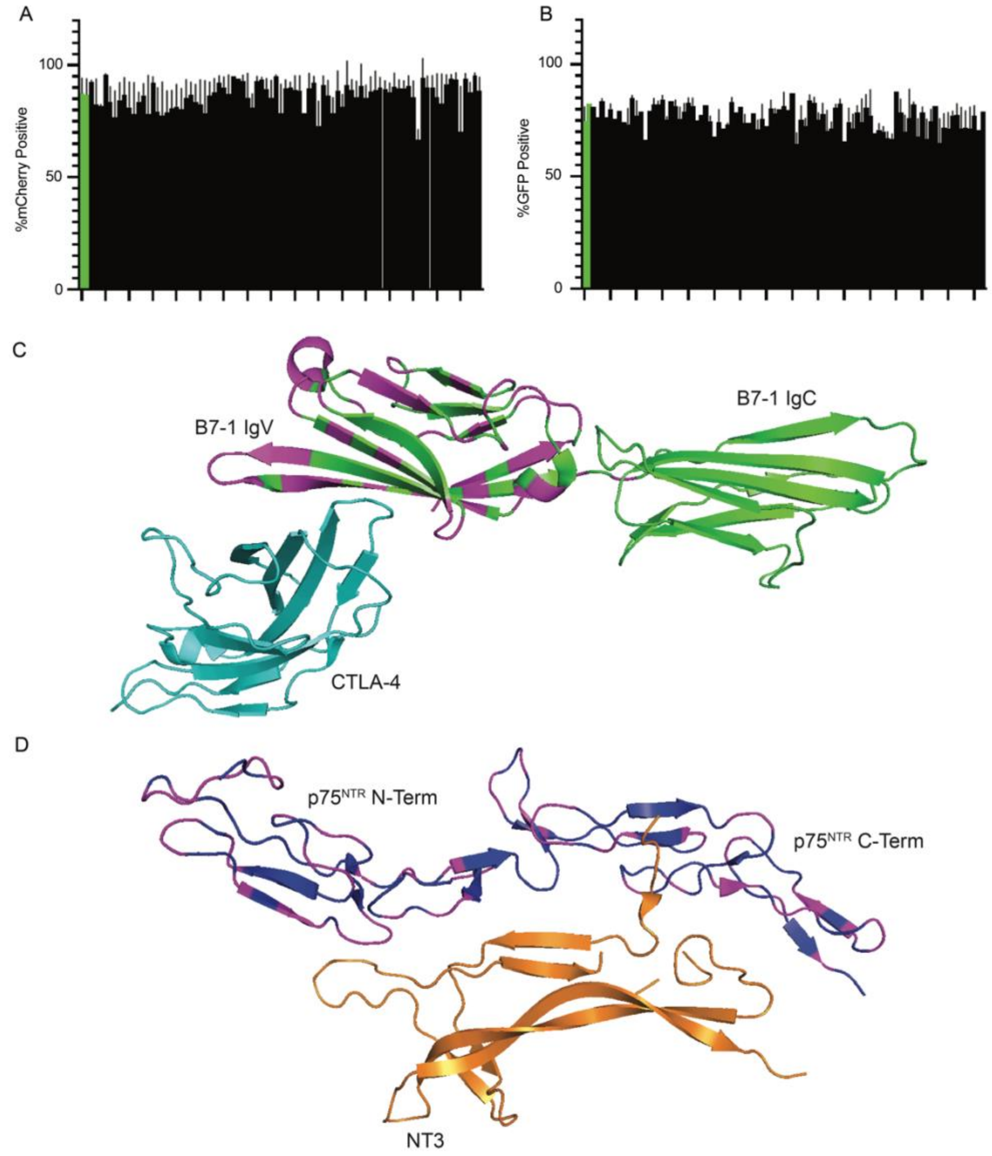

Figure S3: p75^{NTR} and B7-1 Point Mutant Library Expression: A) Average expression of B7-1-mCherry point mutant library for each mutant included in mutagenesis mapping analysis **B)** Average expression of p75^{NTR} - GFP point mutant library for each mutant included in mutagenesis mapping analysis **C)** Mutagenesis coverage of B7-1-mCherry point mutant library **D)** Mutagenesis coverage of p75^{NTR}-GFP point mutant library. See Supplemental table S2 and S3 for information on number of replicates for each data point.

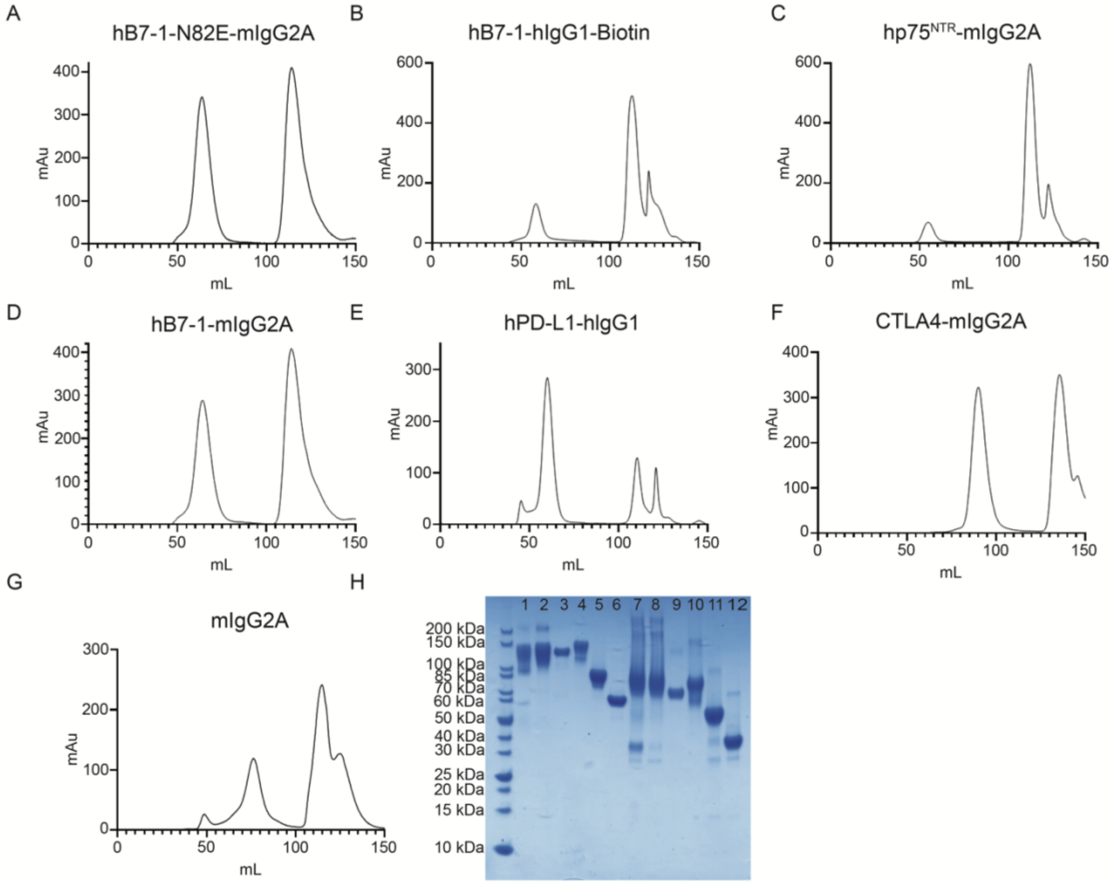

Figure S4: Characterization of Recombinant Proteins: Size exclusion chromatography of purified recombinant protein used in this study. **A)** hB7-1N82E-mIgG2A **B)** hB7-1-hIgG1-biotin **C)** hp75^{NTR}-mIgG2A **D)** hB7-1-mIgG2A **E)** hPD-L1-hIgG1 **F)** hCTLA-4-mIgG2A **G)** mIgG2A - the second peak represents imidazole **H)** SDS-Page analysis of purified proteins from a-g, lanes 1-6 a,c,d,e,f,g under non reducing conditions, lanes 7-12 a,c,d,e,f,g under reducing conditions.

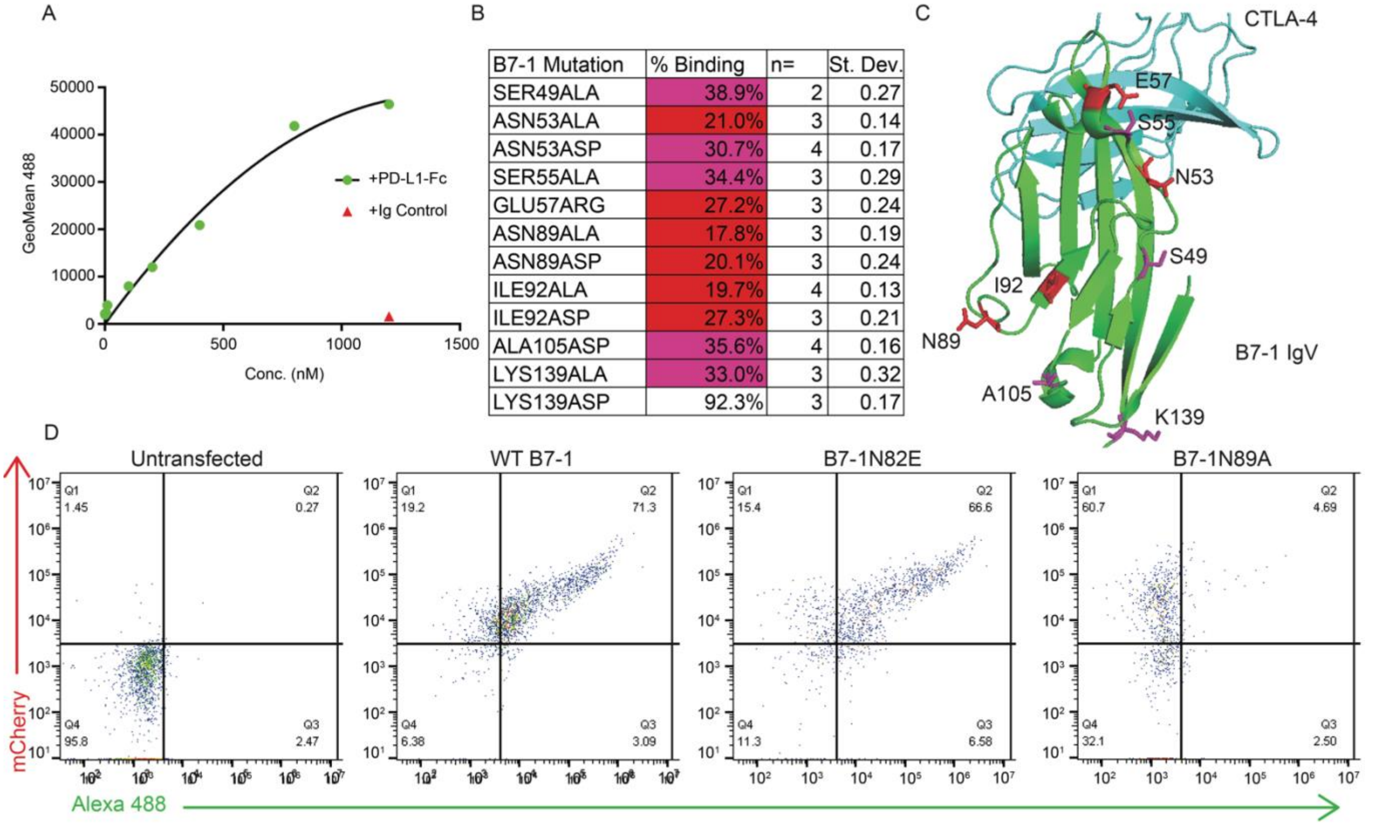

B7-1 Mutation	% Binding	n=	St. Dev.
SER49ALA	38.9%	2	0.27
ASN53ALA	21.0%	3	0.14
ASN53ASP	30.7%	4	0.17
SER55ALA	34.4%	3	0.29
GLU57ARG	27.2%	3	0.24
ASN89ALA	17.8%	3	0.19
ASN89ASP	20.1%	3	0.24
ILE92ALA	19.7%	4	0.13
ILE92ASP	27.3%	3	0.21
ALA105ASP	35.6%	4	0.16
LYS139ALA	33.0%	3	0.32
LYS139ASP	92.3%	3	0.17

Figure S5: B7-1:PD-L1 Binding Interface. The B7-1:PD-L1 binding interface was analyzed by assessing binding of PD-L1-Fc to B7-1 point mutation library**. A)** Representative binding curve showing that PD-L1-Fc binds to B7- 1 expressing cells, binding was detected using and anti Fc 488 labeled antibody and flow cytometry. **B)** B7-1 point mutations drastically reduced binding to PD-L1-Fc **C)** mutant positions for (B) showing that the PD-l1 binding site is on the opposite face of B7-1 as the CTLA-4/CD28 binding interface **D)** representative flow plots showing PD-L1-Fc binding to untransfected cells, WT-B7-1 expressing cells, B7-1N82E expressing cells, or B7- 1N89A expressing cells.

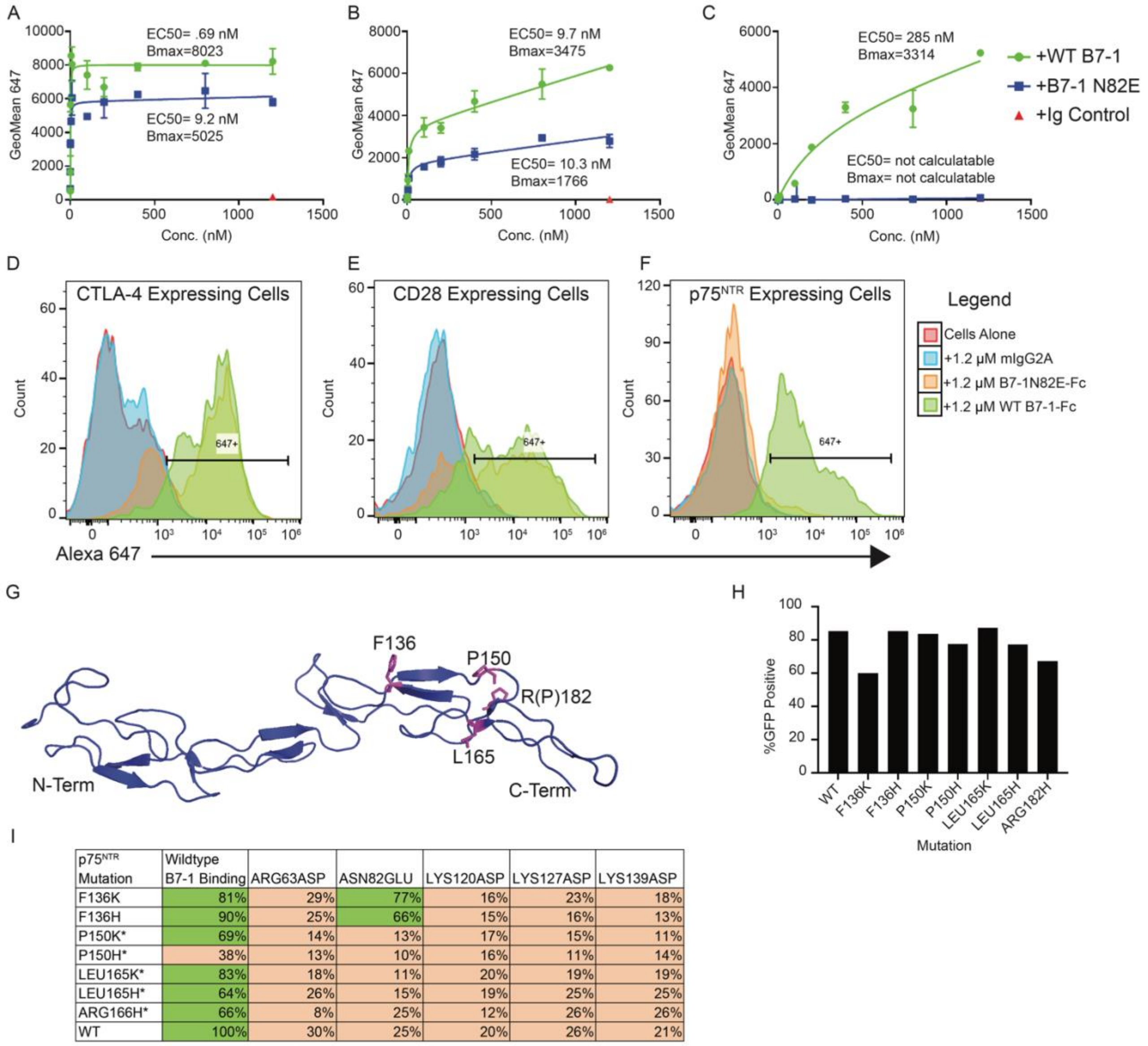

I

p75NTR Mutation	Wildtype B7-1 Binding	ARG63ASP	ASN82GLU	LYS120ASP	LYS127ASP	LYS139ASP
F136K	81%	29%	77%	16%	23%	18%
F136H	90%	25%	66%	15%	16%	13%
P150K*	69%	14%	13%	17%	15%	11%
P150H*	38%	13%	10%	16%	11%	14%
LEU165K*	83%	18%	11%	20%	19%	19%
LEU165H*	64%	26%	15%	19%	25%	25%
ARG166H*	66%	8%	25%	12%	26%	26%
WT	100%	30%	25%	20%	26%	21%

Figure S6: Characterization of B7-1-N82E Mutant: Recombinant B7-1-Fc and B7-1N82E-Fc was purified and titrated onto cells expressing **A)** CTLA-4 **B)** CD28 or **C)** p75^{NTR}, n=4. Binding was detected using an anti-mouse 647 labeled antibody by flow cytometry, calculated EC50 and Bmax are shown. No binding can be detected between B7-1N82E-Fc and p75^{NTR} expressing cells. Representative flow plots of recombinant B7-1-Fc and B7-1N82E-Fc binding to **D)** CTLA-4 cells **E)** CD28 cells and **F)** p75^{NTR} expressing cells are shown. **G)** p75^{NTR} amino acid positions chosen for stabilizing salt bridge screen are shown **H)** representative transfection of salt bridge mutants indicating they express similarly to wild type **I)** results of full stabilizing salt bridge screen showing each pairwise interaction and %-binding compared to wild type, *only screened once. All binding curves were generated by 3 independent experiments, each with one experimental replicate.

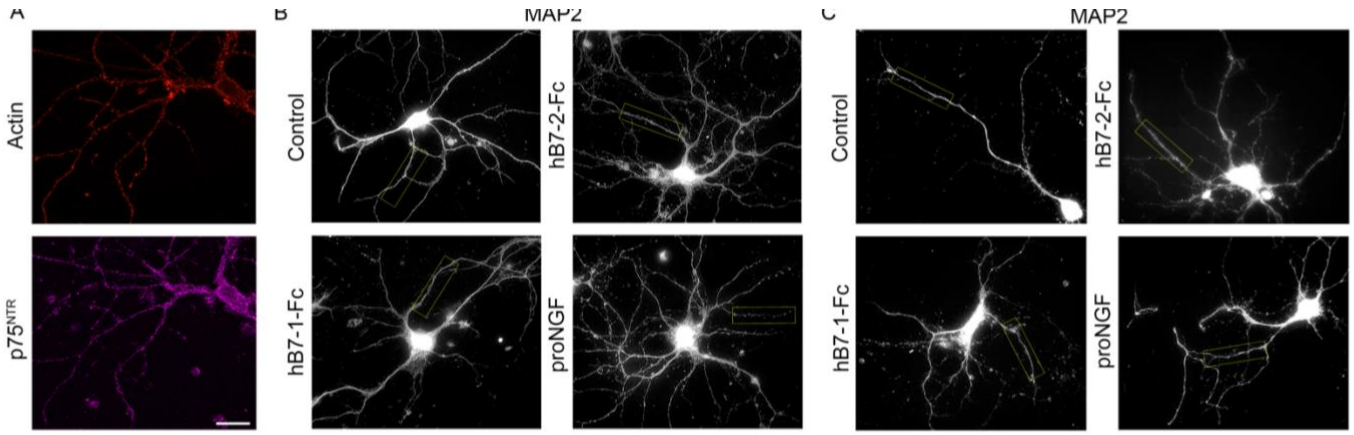

Figure S7. DIV 18 hippocampal neurons express p75^{NTR}. A) Representative IF of Wild-Type hippocampal neurons showing expression of p75^{NTR} at DIV 18. **B)** Representative IF images of Wild-Type hippocampal neurons treated with proNGF (10 nM), hB7-1-Fc (750 nM), or hB7-2-Fc (750 nM) and stained for actin, PSD95, and MAP2, **C)** Representative IF images of *p75*$^{-/-}$ hippocampal neurons treated with proNGF (10 nM), hB7-1-Fc (750 nM), or hB7-2-Fc (750 nM) and stained for actin, PSD95, and MAP2. Boxes show segments used in **Figure 4**. Scale bar = 20 μm. n= 3 independent experiments.

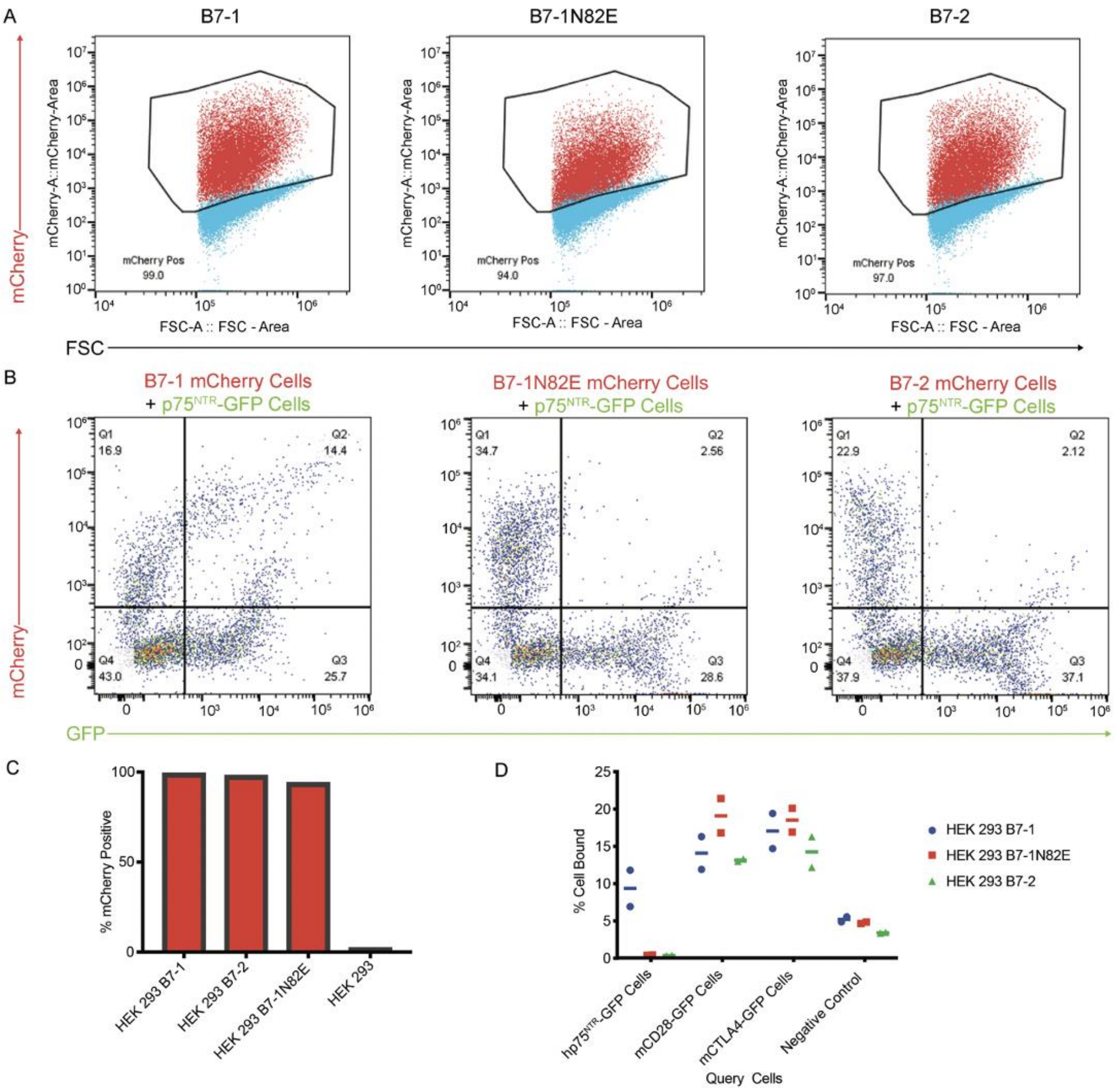

Figure S8: Characterization of Stable B7 cells lines: HEK293 cells were stably transfected to expressed B7-1- mCherry, B7-2-mCherry, or B7-1N82E-mCherry and sorted to generate cells lines expressing similar levels of ligand. **A)** mCherry expression of all three cells lines post sorting **B)** Representative flow plots showing that only HEK293-B7-1-mCherry cell line binds to cells expressing $p75^{NTR}$ **C)** Percentage of stably transfected B7-1, B7-2, or B7-1N82E cells positive for mCherry **D)** quantitation of cell:cell binding between expressed B7-1-mCherry, B7-2-mCherry, or B7-1N82E-mCherry cell lines and cells expressing CTLA-4, CD28, or $p75^{NTR}$ showing that all three lines bind CD28 and CTLA-4 similarly.

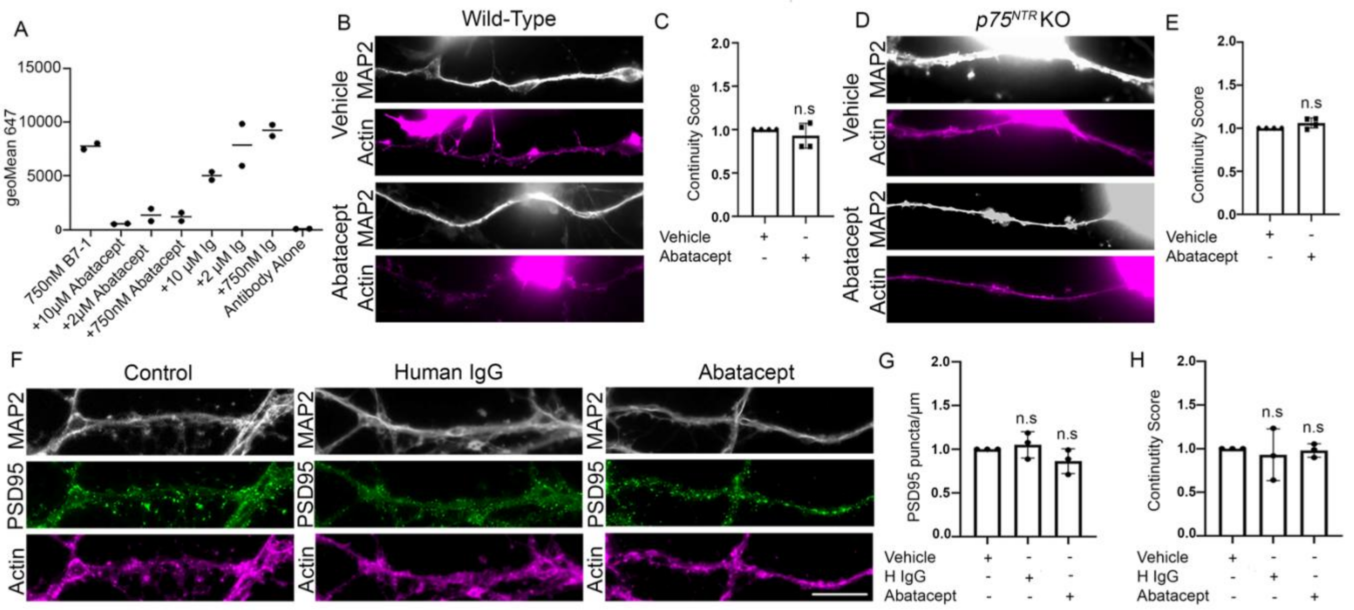

Figure S9. Abatacept (Orencia®) could block the interaction between p75^{NTR} and hB7-1, but the Abatacept (Orencia®) treatment *per se* did not affect the PSD95 density or the morphology of the MAP2 positives neuronal processes. A) 750 nM hB7-1-Fc was incubated with p75^{NTR} expressing cells either alone or with increasing concentrations of Abatacept (Orencia®) or Ig control. 750 nM Abatacept prevent 750 nM hB7-1-Fc from binding to p75^{NTR}-expressing cells. **B)** Representative IF images of Wild-Type hippocampal neurons co-cultured with HEK293 cells treated with or without Abatacept (Orencia®) 375nM. **C)** MAP2 positive dendrites in direct contact with HEK293 cell lines were quantified using Continuity score, t test, t=0.9662. **D)** Representative IF images of *p75*$^{-/-}$ hippocampal neurons co-cultured with HEK293 cells treated with or without Abatacept (Orencia®) 375nM. **E)** MAP2 positive dendrites in direct contact with HEK293 cells were quantified using Continuity score, t test, t=0.0758. n=3 independent experiments in which 12-14 individual neurons were analyzed. **F)** Representative IF images of Wild-Type hippocampal neurons treated with Human IgG (H IgG) or Abatacept (Orencia®) 375nM. After 4 hours of treatment, no differences were found in **G)** PSD95 density (p=0.6385) or in **H)** MAP2 continuity score (p=0.8890) following Human IgG or Abatacept (Orencia®) 375nM treatment. One-way ANOVA, with Dunnett's multiple comparisons test. n=3 independent experiments in which 12-14 individual neurons were analyzed. per treatment per experiment, bar = 20μm.

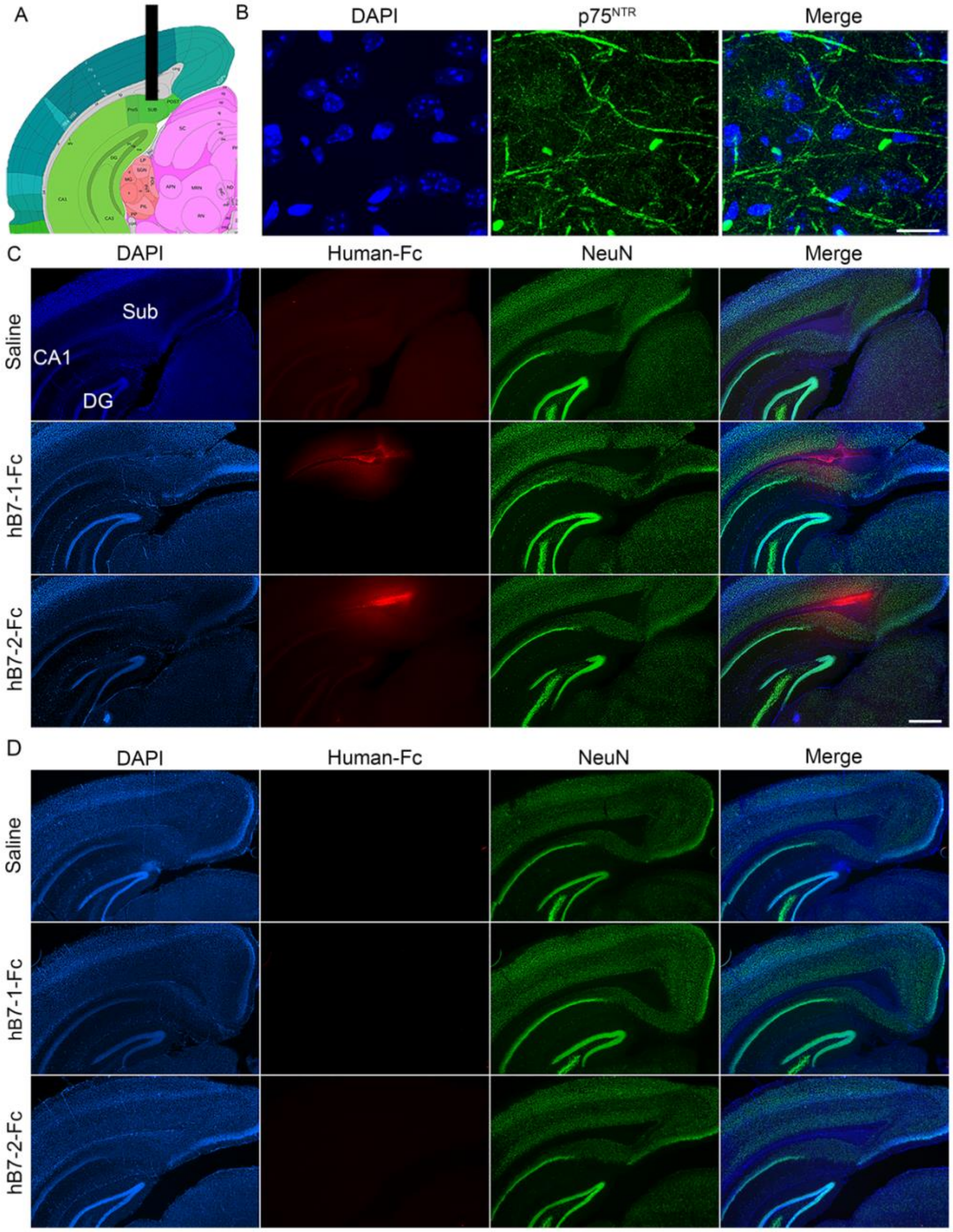

Figure S10 Recombinant hB7-1-Fc and Recombinant hB7-2-Fc injected into the Subiculum could be detected 3h post injection.

A) Representative section of dSubiculum of the hippocampal formation showing injection site[1]. **B)** Representative IF images with neurons Immunostained for p75^{NTR} at P75 (63x), Scale bar: 20 µm. **C)** Representative IF images of Wild-Type dSubiculum (bregma -3.52 mm, AP=-3.52mm, ML=2.5mm, DV=1.75 mm) 3h post injection of hB7-1-Fc (200ng), hB7-2-Fc (200ng) or Saline. hB7-1-Fc and hB7-2-Fc. hB7 proteins presence was confirmed using immunofluorescence. **D)** Representative IF images of Wild-Type dSubiculum (bregma -3.52 mm, AP=-3.52mm, ML=2.5mm, DV=1.75 mm) hB7-1-Fc (200 ng), hB7-2-Fc (200 ng) or Saline 24h post injection. hB7-Fc proteins were not detected in the dSubiculum after 24h. Scale bar: 500µm. Sub: Subiculum, DG: Dentate Gyrus, CA1: *Cornu Amonnis* 1. n=3 independent experiments.

Supplemental Methods:

HEK293 Freestyle Cell culture

HEK293 Freestyle cells were cultured in HEK Freestyle Media (Invitrogen, 12338018) grown at 37° C in a humidified shaking platform incubator (Kuhner, Climo Shaker ISF4-X) with 5% CO_2. For transfection, cells were pelleted at 500xg and resuspended in fresh media. For small-scale (1mL cells at 1x106/mL) transient transfections performed in 24-well non-treated tissue culture plates, 2 μg Polyethylenimine (PEI, Fisher Scientific, AC178571000) was added to 0.5 μg diluted plasmid DNA in a final volume of 100 μL. For large-scale transfections (600mL cells at 1x106/mL) carried out in 2L baffled sterile shake flasks, 2 mg PEI was added to 400 μg diluted plasmid DNA.

Protein Purification:

Seven days post transfection, the media was harvested, 50 mM MES was added to adjust to pH 6.5 and 100 mM Arg-Cl (pH 6.5) was added to enhance solubility. Fc-fusions were subsequently purified by Ni2+His60 chromatography (GE) using a batch binding method (3 mL resin bed volume) followed by gravity flow over a column. The Ni^{2+}His60 resin was washed with 3 column volumes of wash buffer (50 mM MES pH 6.5, 100 mM Arg-Cl, 5 mM imidazole, 150 mM NaCl, 10% Glycerol) and the bound protein eluted with 5 mL the same buffer containing 500 mM imidazole. Nickel column elutes were concentrated and further purified by gel filtration on an S200 Sephadex column (MilliporeSigma, GE29321905) equilibrated with 50 mM MES pH 6.5, 100 mM Arg-Cl, 150 mM NaCl, 10% Glycerol. All recombinant proteins were used within one week of purification or were frozen at -80 C and only thawed one time. Frozen aliquots of protein were utilized but routinely checked for potential aggregation by analytical size chromatography). B7-1N82E-Fc eluted at the same volume as WT B7-Fc when purified using size exclusion chromatography (**Supplemental Figure S4**), indicating the N82E mutation likely does not affect the overall stability of the B7-1 protein.

Site Directed Mutagenesis

For B7-1, positions selected for mutagenesis were based on the crystal structure of complex formed by human B7-1 and human CTLA-4 (PDB: 1I8L). Surface accessible residues in the IgG domain of B7-1 were identified using GetArea(30) (56 positions total). For p75^{NTR}, positions selected for mutagenesis were based on the crystal structure of complex formed by rat p75^{NTR} and human NT3 (PDB: 3BUK). Mutagenesis was attempted such that each chosen position was mutated to an Ala and Asp/Glu/Arg residue. The overall mutagenesis success rate was ~90%, and for some positions not all substitutions (A, D, E, R) were obtained. The sequence validated mutants were expression tested by transient transfection of 1 mL of suspension HEK 293 cells. Only those mutants exhibiting comparable expression to wild type B7-1 or p75^{NTR} and correct plasma membrane localization were subsequently utilized in further binding studies, yielding a final library of 89 B7-1 mutants and 108 p75^{NTR} mutants to assay.

Microbead Cell Flow Cytometry Binding Assay

Microbead Cell Flow Cytometry Binding Assays were performed as previously described with modifications(24). See Supplemental Information for details. For each experiment, 5 uL fluorescent protein A microbeads (either pink or yellow, Spherotech, PAFP-0552-5, PAFP-0558-5) were loaded with a mixture of 10 μg Fc fusion protein of interest (either B7-1 or p75^{NTR}) of in a total volume of 500 uL 1x PBS 0.2% BSA, pH 7.4. The beads were incubated for 30 minutes at room temperature. Beads were washed once by pelleting at 3000g for 15 minutes and resuspended in 500 uL PBS .2% BSA, pH 7.4. Loaded beads were used within 12 hours. For bead:cell binding experiments, 5 uL of beads loaded with either B7-1-Fc, p75^{NTR}-Fc, or Fc control, were incubated with 50,000 Freestyle HEK 293 cells expressing ligands if interest in a total volume of 50 uL for 20 minutes. Bead:HEK cell binding was assessed by flow cytometry on a SONY Spectral Analyzer (SA 3800).

Competition experiments

For Competition experiments, fluorescent protein A beads were loaded and incubated with cells expressing a ligand of interest as described above. CD28-Fc protein was purchased from Biolegend (755706). Competition

was assessed by incubating increasing concentrations of ligand with the bead:cell binding reaction and determining binding of beads to cells by flow cytometry. Percent bound was calculated by divided the geomean fluorescence intensity of a given reaction by the geomean fluorescence of that reaction without competing protein.

Co-Culture Experiments

HEK 293 cells were washed with 10 ml warm neurobasal medium supplemented with B27, 1 mM sodium pyruvate, 6mM Glutamax, 100 U ml^{-1} penicillin-streptomycin and 4 µM cytosine-1-b-D-arabinofuranoside per 100x20 culture dish to prevent HEK 293 overgrowth. HEK 293 cells were triturated without trypsinization, counted, and seeded at a density of 20 x 10^3 per 12 mm glass coverslip with hippocampal neuronal cultures (Wild-Type or *p75*$^{-/-}$). The mixed culture was incubated for 4 hours and fixed with pre-warmed 4% paraformaldehyde / 4% sucrose solution for 15 min at room temperature.

THP-1 cells (ATCC® TIB-202) were differentiated by adding 50ng/mL PMA (Thermo J63916.LB0) and incubated for 24 hours in a 37ºC humidified CO_2 incubator. After 24h the media was replaced, and the cells were kept for another 24h. The media was replaced with fresh RPMI media +/- LPS at 1mg/ml. The cells were incubated for another 24h. After 24h stimulation, the cells were washed 3 x 30 min and were detached by scraping, then counted and seeded at a density of 20 x 10^3 per 12 mm glass coverslip with wild-type hippocampal neuronal cultures. The mixed culture was incubated for 4 hours and fixed with pre-warmed 4% paraformaldehyde / 4% sucrose solution for 15 min at room temperature. For flow cytometry, differentiated and stimulated THP-1 cells (100K) were harvested in 1X PBS and 0.2% BSA an stained with 0.5 µg of Alexa 488 anti-human CD80 (B7-1) antibody (Biolegend 305214). Cells were gated for positive hB7-1 expression based on unstained THP-1 populations.

Immunocytochemistry

For immunocytochemistry of the hippocampal neuronal cultures and mixed culture assays, cells were permeabilized and blocked with 3% fetal bovine serum, 3% BSA, and 0.1% Triton X-100 in phosphate buffered saline for 15 minutes at room temperature. Primary antibodies were applied overnight at 4ºC, and subtype-specific Alexa fluorescent secondary antibodies were added for 40 minutes at room temperature. Coverslips were

mounted with ProLong Gold antifade reagent (Invitrogen; P36934). The primary and secondary antibodies and their sources are listed in the Reagents supplementary table (Table XX) anti-PSD-95 (Abcam; ab2723), anti-MAP2 (Abcam; ab92434), anti-Bassoon (Enzo, ADI-VAM-PS003-D- Clone SAP7F407), anti-GluR1 (Invitrogen, PA1-46151) and anti-p75^{NTR} (R&D Systems; AF1157). Secondary antibodies were Alexa Fluor antibodies (Life Technologies, Norwalk, CT, USA), except for the 405nm fluorescent DyLight (Jackson ImmunoResearch Laboratories, West Grove, PA, USA; 102649-302). For visualization of actin cytoskeleton, Alexa Fluor 546 and 647 phalloidin (Life Technologies, Thermo Fisher Scientific; A22283 & A30107) were used.

<u>MAP2 Quantitation</u>

Data was batch analyzed in Python using the following lines of code:

```
import numpy as np

import os

# Change these for appropriate
folders
ROOT_FOLDER = "Target
Folder" OUTPUT_FILE_NAME
= "output.csv" DATA_FOLDER
= os.path.join(ROOT_FOLDER,
"data")
with open(os.path.join(ROOT_FOLDER, OUTPUT_FILE_NAME), "w") as newfile:
# Write header
newfile.write("filename,area_under_curve,area_total,continuity_score (%)\n")
# Loop through each file for the calculation
```

```
filenames = os.listdir(DATA_FOLDER)

for filename in filenames:

data = np.genfromtxt(

os.path.join(DATA_FOLDER, filename),

delimiter=',',

skip_header=1

   )

   x = data[:,0]

   y = data[:,1]

max_x = max(data[:,0])

# Get calculations

area_under_curve = np.trapz(y, x)

area_total = max_x * 255

continuity_score = area_under_curve / area_total * 100

                        newfile.write(

                        filename+","+

                                    str(area_under_curve)+","+

                                                str(area_total)+","

                                                +

                                    str(continuity_score)+"\n")
```

Brightfield and Fluorescence Microscopy

Recombinant protein assay images were acquired on an inverted fluorescent Nikon Eclipse Ti2-E microscope equipped with Andor Zyla 5.5 sCMOS camera and a Lumencor SOLA-SE II light engine. The objectives used were CFI Nikon PlanApo Lambda 20x with 0.75 NA and CFI Nikon Plan Apo Lambda 60x oil lenses with 1.40NA and a 1.5X adapter. Acquisition settings were kept consistent across all conditions within a given experiment. For high magnification, mixed culture assay images were taken on a Zeiss Cell Observer SD confocal with a Yokagawa CSU-X1 spinning disk, Plan-Apochromat 63X/1.4 M27 objective paired with a 1.2X adapter to a Photometrics Evolve 512 EMCCD camera was used for image acquisition. The laser lines used were 405nm, 488nm, 561nm and 639nm.

Golgi Staining

Three hours following stereotactic injection of hB7-1-Fc, hB7-2-Fc or diluent control mice were euthanized using euthasol (0.1 ml/10 g body weight). The brains were rapidly removed and were processed following modified Golgi-Cox protocol. Sections were covered by coverslips with DPX mounting medium, air-dried for 2-3 days and imaged under the light microscope. Pyramidal neurons for spine analysis were chosen from the dSubiculum. Neurons were analyzed by the experimenter blinded to the conditions. The quantification was performed on the apical dendrite (segment 50-150μm away from cell soma).

Statistics:

Quantification and Statistical Analysis

For quantitative assessment of synaptic puncta, the PSD95/ Bassoon/GluR1 channels were analyzed and processed with FIJI software. Secondary and tertiary dendritic branches were selected blind, based on the actin and MAP2 channels. Background was subtracted and a threshold was applied. PSD95/Bassoon/GluR1 puncta on secondary and tertiary dendritic branches were counted. Lastly, the length of the branch was measured, to assess

the density. Experiments were performed at least with triplicate replicates per condition and independently replicated 3 times with similar results. All data was acquired and analyzed in a blinded fashion, and experiments were repeated by different individuals to ensure reproducibility. All data were analyzed with GraphPad Prism 5.0 software (San Diego, CA, USA). Experimental data were statistically analyzed by one-way ANOVA with Bonferroni *post hoc* tests to control for multiple comparisons (for three or more group comparisons), or two-way ANOVA with Bonferroni post hoc tests (to assess statistical significance between means), as indicated within individual figure legends. In figures, asterisks denote statistical significance marked by *$p < 0.05$, **$p < 0.01$, ***$p < 0.001$, and ‘‘n.s.’’ indicates no statistical significance. All statistical parameters are presented as means ± SEM (Standard Error of Mean).

Statistical Information

All data were analyzed with GraphPad Prism 5.0 software (San Diego, CA, USA). For quantitative assessment of synaptic puncta, the PSD95 channel was analyzed and processed with FIJI software. Secondary and tertiary dendritic branches were selected blind, based on the actin and MAP2 channels. Background was subtracted and a threshold was applied. PSD95 puncta on secondary and tertiary dendritic branches were counted. For MAP2 Continuity Score quantification see **MAP2 Quantitation** section. Golgi stained brains: dSubiculum pyramidal neurons were selected and the apical dendrite segment 50-150µm away from the soma were used for quantification. All statistical parameters are presented as means ± SEM (Standard Error of Mean).

Figure 1. Panel D: B7-1 homologue screening vs. human $p75^{NTR}$: n=3,One way ANOVA summary: F=108.2, p<.0001 (***). Tukey's multiple comparisons test: hB7-1 vs. mCherry p<.0001, squirrel monkey B7-1 vs. mCherry p<.0001, white sided dolphin B7-1 vs. mCherry p=.9997, camel B7-1 vs. mCherry p=.9997, pangolin B7-1 vs. mCherry p>.9999, mouse B7-1 vs. mCherry p=.9994, rat B7-1 vs. mCherry p>.9999, sperm whale B7-1 vs. mCherry p=.9994. B7-1 homologue screening vs. mouse $p75^{NTR}$: n=3,One way ANOVA summary: F=35.48, p<.0001 (***). Tukey's multiple comparisons test: hB7-1 vs. mCherry p<.0001, squirrel monkey B7-1 vs.

mCherry p=0001, white sided dolphin B7-1 vs. mCherry p=.9978, camel B7-1 vs. mCherry p=.9997, pangolin B7-1 vs. mCherry p=.9999, mouse B7-1 vs. mCherry p=.9994, rat B7-1 vs. mCherry p=.9999, sperm whale B7-1 vs. mCherry p=.9997. B7-1 homologue screening vs. rat p75^{NTR}: n=3,One way ANOVA summary: F=27.78, p<.0001 (***). Tukey's multiple comparisons test: hB7-1 vs. mCherry p<.0001, squirrel monkey B7-1 vs. mCherry p<0001, white sided dolphin B7-1 vs. mCherry p=.9489, camel B7-1 vs. mCherry p=.9932, pangolin B7-1 vs. mCherry p=.7965, mouse B7-1 vs. mCherry p=.9999, rat B7-1 vs. mCherry p=.8642, sperm whale B7-1 vs. mCherry p=.5264. B7-1 homologue screening vs. GFP: n=3,One way ANOVA summary: F=.7625, p=.6391. Tukey's multiple comparisons test: hB7-1 vs. mCherry p=.9997, squirrel monkey B7-1 vs. mCherry p=.9975, white sided dolphin B7-1 vs. mCherry p=.9968, camel B7-1 vs. mCherry p=.9949, pangolin B7-1 vs. mCherry p=.9181, mouse B7-1 vs. mCherry p=.9994, rat B7-1 vs. mCherry p=.9643, sperm whale B7-1 vs. mCherry p=.9384. B7-1 homologues rat and mouse n=2 for all conditions as indicated in figure.

Figure 3. Panel D: p75^{NTR}F136K,F136H screening against B7-1 point mutations: n=6, One way ANOVA summary: F=59.07, p<.0001 (***). Tukey's multiple comparisons test: wt p75^{NTR} vs. p75^{NTR}F136K: p<.0001, wt p75^{NTR} vs. p75^{NTR}F136H: p<.0001, p75^{NTR}F136K vs. p75^{NTR}F136H: p=.1541.

Figure 4. Panel B. Wild-type Hippocampal neurons culture: n=3, ANOVA summary: F= 11.53, *p*=0.0028; Dunnett's multiple comparisons test: Control vs. proNGF, *p*=0.0115 (*); Control vs. hB7-1-Fc, *p*=0.0020 (**); Control vs. hB7-2-Fc, *p*=0.5095. Panel D: *p75$^{(-/-)}$* Hippocampal neuron culture: n=3, ANOVA summary: F= 0.2280, *p*= 0.8743; Tukey's multiple comparisons test: Control vs. proNGF *p*=0.9183; Control vs. hB7-1-Fc *p*= 0.9133; Control vs. hB7-2-Fc *p*= 0.9992. Panel F: Bassoon puncta density Wild-type Hippocampal neurons culture: p=0.0073, F= 12.45, Control vs hB71 *p*=0.0066, hB72 vs hB71 *p*=0.036, Control vs hB72: 0.3306. Panel G: GluR1 puncta density Wild-type Hippocampal neurons culture: p=0.0015, F=22.96. Control vs hB71: *p*=0.0029, hB72 vs hB71 *p*=0.0024, Control vs hB72: p=0.9761. 13-15 dendritic segments per condition, per experiment. N=3.

Figure 5. Panel B: Wild-type Hippocampal neurons and HEK 293 B7-expressing cells mixed culture: *PSD puncta/µm*: n=3, one way ANOVA summary: F= 55.39, *p*= 0.0001 (***); Tukey's multiple comparisons test:

hB7-1 vs. hB7-1N82E *p*=0.0003 (***); hB7-1 vs. hB7-2 *p*=0.0002 (***), hB7-1N82E vs. hB7-2 *p*=0.6468. Panel C: *MAP2 Continuity Score*: n=3, one-way ANOVA summary: F= 36.55, *p*=0.0004 (***); Tukey's multiple comparisons test: hB7-1 vs. hB7-1N82E *p*=0.0007 (***); hB7-1 vs. hB7-2 *p*=0.0008, hB7-1N82E vs. hB7-2 *p*=0.9826.

Panel E: Bassoon puncta density: p<0.0001, F=190.1, hB72 vs hB71 *p*<0.0001, hB72 vs hB71N28E *p*=0.7267, hB71N28E vs hB71: p=<0.0001. 13-15 dendritic segments per condition per experiment. N=3.

Figure 6. Panel B: Wild-type Hippocampal neurons culture and HEK 293 B7-expressing cells Abatacept treatment: *MAP2 Continuity Score*: n=3, two-way ANOVA summary: Source of Variation: Interaction *p* <0.0001 (****), DF 2, F (DFn, DFd)= F (2, 12) = 26.40. Tukey's multiple comparisons test hB7-1:None vs. hB7-1:Orencia 375nM, hB7-1:None vs. hB7-1N82E:None, hB7-1:None vs. hB7-1N82E:Orencia 375nM, hB7-1:None vs. hB7-2:None, B7-1:None vs. hB7-2:Orencia 375nM all *p*<0.0001. hB7-1:Orencia 375nM vs. hB7-1N82E:None p= 0.1382; hB7-1:Orencia 375nM vs. hB7-1N82E:Orencia 375nM *p*= 0.1396; hB7-1:Orencia 375nM vs. hB7-2:None *p* >0.9999; hB7-1:Orencia 375nM vs. hB7-2:Orencia 375nM *p*= 0.3477; hB7-1N82E:None vs. hB7-1M:Orencia 375nM *p* >0.9999; hB7-1N82E:None vs. hB7-2:None *p*= 0.1316; hB7-1N82E:None vs. hB7-2:Orencia 375nM *p*= 0.9859; hB7-1N82E:Orencia 375nM vs. hB7-2:None *p*= 0.1330; hB7-1N82E:Orencia 375nM vs. hB7-2:Orencia 375nM *p*= 0.9865; hB7-2:None vs. hB7-2:Orencia 375nM *p*= 0.3339. Panel D: *p75*^{NTR} KO Hippocampal neurons culture and HEK 293 B7-expressing cells mixed culture. *MAP2 Continuity Score*: n=3, two-way ANOVA summary: Interaction *p*=0.1860, F (4, 18) =1.736. Tukey's multiple comparisons test B7-1:None vs. B7-1:Orencia 375 nM, *p*=0.9715, B7-1:None vs. hB7-1N82E:None *p*=0.9937, B7-1:None vs. hB7-1N82E:Orencia 375 nM *p*=0.8085, B7-1:None vs. B7-2:None *p*=0.9973, B7-1:None vs. B7-2:Orencia 37 5nM *p*=0.6998, B7-1:Orencia 375 nM vs. hB7-1N82E:None *p*=0.6158, B7-1:Orencia 375 nM vs. hB7-1N82E:Orencia 375 nM p=0.9998, B7-1:Orencia 375 nM vs. B7-2:None *p*=0.6752, B7-1:Orencia 37 5nM vs. B7-2:Orencia 375nM *p*=0.1700, hB7-1N82E:None vs. hB7-1N82E:Orencia 375 nM *p*=0.3357, hB7-1N82E:None vs. B7-2:None *p*>0.9999, hB7-1N82E:None vs. B7-2:Orencia 375 nM *p*=0.9878, hB7-1N82E:Orencia 375nM vs. B7-2:None *p*=0.3853, hB7-1N82E:Orencia 375 nM vs. B7-2:Orencia 375nM

p=0.0687, B7-2:None vs. B7-2:Orencia 375 nM p=0.9774. 13-15 dendritic segments per experiment, per condition. N=3.

Figure 7. THP-1 cells treated with/without LPS induced MAP2 positive neuronal processes degradation. Panel D: p=0.0003, F = 21.59. THP1-LPS vs THP1+LPS: p=0.0017; THP1+LPS+Abatacept vs THP1+LPS, p=0.0028; THP-1-LPS+Abatacept vs THP1+LPS, p=0.0003. n=3, 10-15 dendritic segments per condition per experiment.

Figure 8. Panel B: Stereotactic injections B7 proteins (100) Golgi stained Wild-Type brains. Time point: 3hs, n=4, ANOVA summary F= 8.627, p= 0.0081 (**), Tukey's multiple comparisons test Saline vs. B7-1 100 ng, p= 0.0268 (*), Saline vs. B7-2 100 ng, p= 0.7662, B7-1 100 ng L vs. B7-2 100 ng, p= 0.0091 (**). Panel D: Stereotactic injections B7 proteins (100 ng) Golgi stained *p75*$^{(-/-)}$ brains. Time point: 3 hr, n=4, 15-20 dendritic segments per condition, ANOVA summary F= 3.418, p= 0.0786, Tukey's multiple comparisons test Saline vs. B7-1 100 ng p= 0.3099, Saline vs. B7-2 100 ng, p= 0.0677, B7-1 100 ng vs. B7-2 100 ng, p= 0.5746. Panel F: Stereotactic injections B7 proteins (200 ng) Golgi stained Wild-Type brains. Time point: 3hs, n=4, 15-20 dendritic segments per condition, ANOVA summary F= 15.13, p= 0.0013 (**), Tukey's multiple comparisons test Saline vs. B7-1 200 ng, p= 0.0243 (*), Saline vs. B7-2 200 ng, p= 0.068. Panel H: Stereotactic injections B7 proteins (200ng) Golgi stained Wild-Type brains. Time point: 24 hr, n=3, ANOVA summary F= 44.79, p= 0.0002 (**), Tukey's multiple comparisons test Saline vs. B7-1 200 ng p= 0.0003 (*), Saline vs. B7-2 200 ng, p= 0.4607.

Supplemental Figure S9. Panel G: Wild-type Hippocampal neurons culture: PSD puncta/µm n=3, one-way ANOVA: F= 0.7227, *p*= 0.6385. Dunnett's multiple comparisons test Control vs. Human IgG 37 5nM *p*=0.9997, Control vs. Abatacept 375 nM *p*=0.9775. N=3, 15 dendritic segments per condition Panel H: *MAP2 Continuity Score*: n=3, one-way ANOVA summary: F= 0.1200, *p*= 0.8890. Dunnett's multiple comparisons test Control vs. Human IgG 375nM *p*=0.8534; Control vs. Abatacept 375nM *p*=0.9867. Panel C: Wild-Type Hippocampal neurons culture and HEK 293 mixed culture. Unpaired t test, Two-tailed, t=0.9662, df=6, *p*=0.3713. Panel E: *p75(*$^{-/-)}$ Hippocampal neurons culture and HEK 293 mixed culture. Unpaired t test, Two-tailed, t=2.143, df=6, t=0.0758.

N=4, 13-20 dendritic segments per condition.

References

1

<https://atlas.brainmap.org/atlas?atlas=602630314#atlas=602630314&plate=576986971&structure=502&x=8128&y=3984&zoom=-4&resolution=16.00&z=3> (
